# Development of NHAcGD2/NHAcGD3 conjugates of bacteriophage MX1 virus-like particles as anticancer vaccines†

**DOI:** 10.1039/d3ra08923a

**Published:** 2024-02-19

**Authors:** Qingyu Zhao, Xuefei Huang, Xuanjun Wu

**Affiliations:** a National Glycoengineering Research Center and Shandong Key Laboratory of Carbohydrate Chemistry and Glycobiology, NMPA Key Laboratory for Quality Research and Evaluation of Carbohydrate-based Medicine, Shandong University Qingdao Shandong 266237 China xuanjun@sdu.edu.cn; b Departments of Chemistry and Biomedical Engineering, Institute for Quantitative Health Science and Engineering, Michigan State University East Lansing Michigan 48824 USA

## Abstract

The successful development of an anticancer vaccine will be a giant leap forward in cancer prevention and treatment. Herein, the bacteriophage MX1 coat protein virus-like particles (MX1 VLPs) have been conjugated with 9NHAc-GD2 (NHAcGD2) to obtain a MX1-NHAcGD2 conjugate. Intriguingly, vaccinating against this conjugate produced a robust anti-NHAcGD2 IgG response in mice, with an average IgG titer of over 3 million. More interestingly, antibodies induced by the MX1-NHAcGD2 conjugate bound well to IMR-32 neuroblastoma cells and had potent complement-dependent cytotoxic (CDC) effects on IMR-32 cells. Inspired by the superiority of the 9NHAc-GD2 antigen, we also designed another 9NHAc-modified ganglioside antigen, 9NHAc-GD3 (NHAcGD3), to overcome the hydrolytic instability of 9-*O*-acetylated-GD3. By coupling NHAcGD3 with MX1 VLP, the MX1-NHAcGD3 conjugate was constructed. Strikingly, vaccination of MX1-NHAcGD3 elicited high anti-NHAcGD3 IgG antibodies, which effectively recognized human malignant melanoma SK-MEL-28 cells and had a significant CDC effect against this cell line. This study provides novel MX1-NHAcGD2 and MX1-NHAcGD3 conjugates with broad clinical translational prospects as promising anticancer vaccines.

## Abstract 2

As a novel class of VLP vaccine carrier, the bacteriophage MX1 virus-like particle (VLP) is used for the first time to construct MX1-NHAcGD2 and MX1-NHAcGD3 conjugates. These conjugates can be exciting leads for anticancer vaccines.

## Graphical abstract

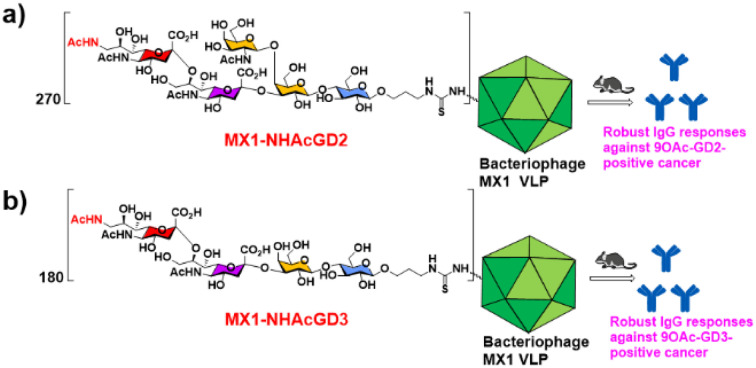

Cancer is a major human disease with high fatality. Globally, there were 19.3 million new cases of cancer in 2020, including nearly 10 million deaths. The number of cancer cases worldwide is expected to reach 28.4 million by 2040.^1^ These alarming data urgently require the development of effective cancer treatments. Commonly used cancer treatment methods include chemotherapy,^2,3^ radiotherapy,^4^ phototherapy,^5–7^ and immunotherapy,^8–11^ of which immunotherapy is attractive because it can call on the immune system to fight cancer cells. As a promising modality of cancer immunotherapy, vaccine immunization can provide long-term protection to the host with few side effects.^12–15^ Therefore, developing effective and safe anticancer vaccines is crucial.

Antigens are one of the indispensable components of vaccines. As one fantastic class of antigens, tumor-associated carbohydrate antigens (TACAs) include Tn, Tf, STn, Globo-H, GM2, GD2, GD3, *etc*,^16^ among which GD2 and GD3 gangliosides are a class of glycosphingolipids with two sialic acid residues linked to lactosylceramides.^17^ They are widely expressed in most cancers of neuroectodermal or mesodermal origin, including melanoma, neuroblastoma and sarcoma. Nevertheless, GD2 and GD3 are also distributed in normal tissues, leading to adverse side effects. For example, treating patients with high-dose GD2 monoclonal antibodies results in dose-dependent acute and/or chronic toxicity due to high expression of GD2 in peripheral nerves.^18^ To overcome side effects, more specific target antigens should be explored.

9-*O*-acetylation is a common natural modification on sialic acid, which impacts many biological phenomena, such as microbial and host interactions.^19^ Compared with GD2 and GD3 antigens, 9-*O*-acetyl-GD2 (9OAc-GD2) and 9-*O*-acetyl-GD3 (9OAc-GD3) are a class of tumor target antigens with higher specificity. 9OAc-GD2 is strongly expressed on the surface of many cancer cells, including neuroblastoma,^20^ glioblastoma,^21^ and breast cancer;^22^ meanwhile, 9OAc-GD3 is overexpressed in melanoma,^23^ glioblastoma,^24^ breast cancer,^25^ and small cell lung cancer.^26^ Since 9OAc-GD2 and 9OAc-GD3 are rarely expressed in normal tissues, antibodies based on these two antigens or antibodies produced by vaccines are highly specific. However, developing vaccines based on 9-OAc-GD2/9-OAc-GD3 is challenging due to the hydrolytic instability of O-acetylated-GD2/or-GD3. To address this challenge, we recently reported a stable 9NHAc-GD2 (NHAcGD2) antigen that mimics the 9OAc-GD2 antigen.^27^ It has been coupled to the bacteriophage Qβ virus-like particle (VLP) to produce the Qβ-NHAcGD2 conjugate, which elicited robust anti-NHAcGD2 IgG antibodies.^27^ The results show that NHAcGD2 is a tumor target antigen with high specificity and indicate the importance of Qβ VLP conjugation in enhancing the anti-NHAcGD2 IgG response.

Building on the above work, we aim to establish more vaccines based on NHAcGD2, such as developing NHAcGD2 conjugates with a new carrier. This is because as the variety of vaccines under development increases, more and more conjugate vaccines share the same carrier; for example, many reported vaccine candidates contain Qβ VLP.^27–37^ The concomitant use of these vaccines may lead to high levels of pre-existing antibodies against the carrier, possibly suppressing the immune responses against new conjugate vaccines sharing the same carrier moiety.^38,39^ Therefore, establishing a new carrier is valuable for expanding the arsenal of vaccines.

In this work, we have begun investigating a novel carrier, the coat protein (CP) VLP of Enterobacteria phage MX1 (a strain of Qβ virus), which has never been used in TACA-based vaccines. The pET-28-MX1-CP plasmid was constructed according to the gene ID 1261502. Strikingly, we achieved a high yield (∼80 mg L^−1^) in preparing MX1 VLPs. Then, we investigated this carrier for NHAcGD2 conjugate vaccine development (Scheme 1a). Intriguingly, MX1-NHAcGD2 elicited high levels of anti-NHAcGD2 IgG responses. Inspired by the superiority of the NHAcGD2 antigen, we envision 9NHAc-GD3 (NHAcGD3) as a promising antigen to mimic 9OAc-GD3. As such, we synthesized the NHAcGD3 antigen and conjugated this new antigen with MX1 VLP to produce MX1-NHAcGD3 conjugate (Scheme 1b). Encouragingly, vaccinating mice with MX1-NHAcGD3 elicited potent anti-NHAcGD3 IgG responses.

**Scheme 1:**
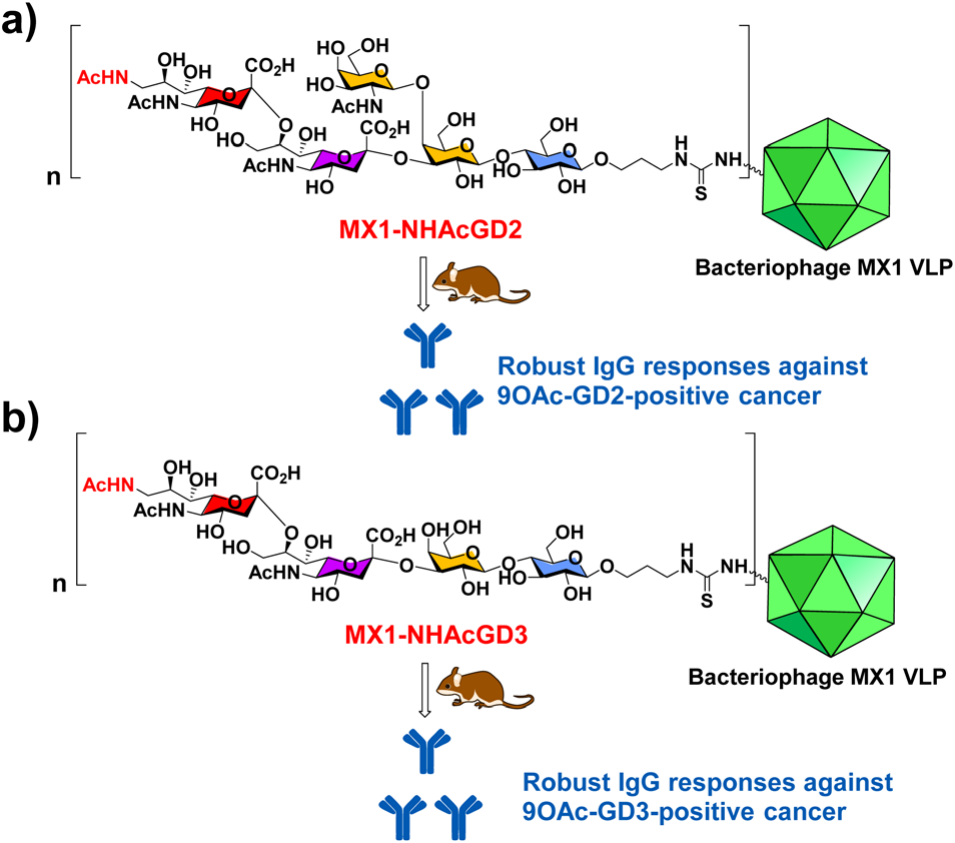
Schematic diagram of the proposed (a) MX1-NHAcGD2 and (b) MX1-NHAcGD3 conjugates for eliciting robust anticancer IgG responses.

## Results and discussion

### Preparation and characterization of MX1 VLP

The pET28-MX1-CP plasmid was constructed according to the gene ID 1261502 and transformed into BL21 (DE3) competent cells. Isopropyl β-d-1-thiogalactopyranoside (IPTG) was used to induce the expression of MX1 VLP. Afterward, the MX1 VLP-expressing bacteria were collected for ultrasonic disruption. The lysate is then subjected to purification steps, including PEG8000 precipitation, chloroform/*n*-butanol organic extraction, and sucrose gradient centrifugation. After purification, the Bradford assay was used to quantify total protein content, using bovine serum albumin (BSA) as a standard. Excitingly, the yield of MX1 VLP is ∼80 mg L^−1^. Previous studies have shown that the yield of Qβ VLP is approximately 30 mg L^−1^,^40,41^ which is consistent with the results we have obtained by multiple expressions of Qβ VLP in the past. The very high yield of MX1 VLP facilitates the large-scale preparation of MX1-TACA conjugate vaccines for clinical translation in the future.

**Fig. 1:**
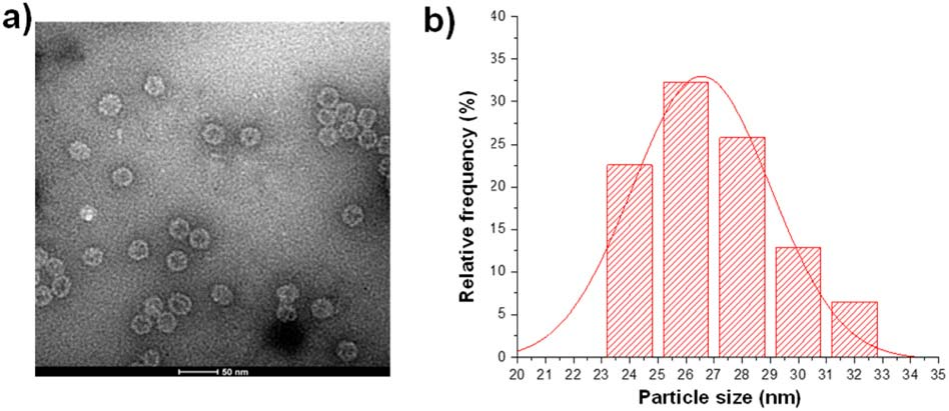
(a) TEM image of the bacteriophage MX1 coat protein virus-like particles (MX1 VLPs). (b) The average diameter of MX1 VLP is 26 nm, as determined by nano-measure software.

The purified MX1 VLP was characterized by size-exclusion HPLC (SEC), SDS polyacrylamide gel electrophoresis (SDS-PAGE), MALDI-TOF mass spectrometry (MS), transmission electron microscopy (TEM), and dynamic light scattering (DLS). SEC and SDS-PAGE results showed good purity of the obtained MX1 VLP (Fig. S1 and S2, ESI†). The MALDI-TOF MS result showed that the molecular weight of the MX1 subunit was 14.1 kDa (Fig. S3, ESI†). TEM showed the nanostructure of MX1 with a mean diameter of 26 nm (Fig. 1), which was almost consistent with the DLS result (Fig. S4, ESI†). MX1's VLP structure will confer its ability to present TACA, such as NHAcGD2, in a highly ordered manner,^42^ thereby eliciting potent anti-TACA IgG antibodies.

### Synthesis of NHAcGD2-isothiocyanate (NCS) 1, MX1-NHAcGD2 conjugate 2, CRM197-NHAcGD2 conjugate 3, and MX1-GD2 conjugate 4

The chemoenzymatic synthesis of NHAcGD2-NCS 1 began with LacβProN_3_ S1 ^43^ (Scheme S1, ESI†), which was incubated with Neu5Ac, cytidine-5′-triphosphate (CTP) in Tris–HCl buffer (100 mM, pH 8.5) containing MgCl_2_, followed by the addition of *Neisseria meningitidis* CMP-sialic acid synthetase (NmCSS) and *Pasteurella multocida* α2,3-sialyltransferase (PmST1) to form GM3-N_3_ S2 in a yield of 92%. Then, S2 was treated with *Campylobacter jejuni* α2,8-sialyltransferase (CjCstII) and NmCSS in the presence of 9NHAc-Neu5Ac S3,^27^ CTP and MgCl_2_, resulting in NHAcGD3-N_3_ S4 in a yield of 81%. By the addition of UDP-GalNAc and *Campylobacter jejuni* β1,4 *N*-acetylgalactosaminyltransferase (CjCgtA) in Tris–HCl buffer (100 mM, pH 7.5) with MgCl_2_, S4 was converted to NHAcGD2-N_3_ S5 in a yield of 83%, followed by Pd/C-H_2_ reduction and isothiocyanate formation producing NHAcGD2-NCS 1. Nuclear magnetic resonance (NMR) spectroscopy and MS were used to characterize the final product and intermediate compounds.

**Scheme 2:**
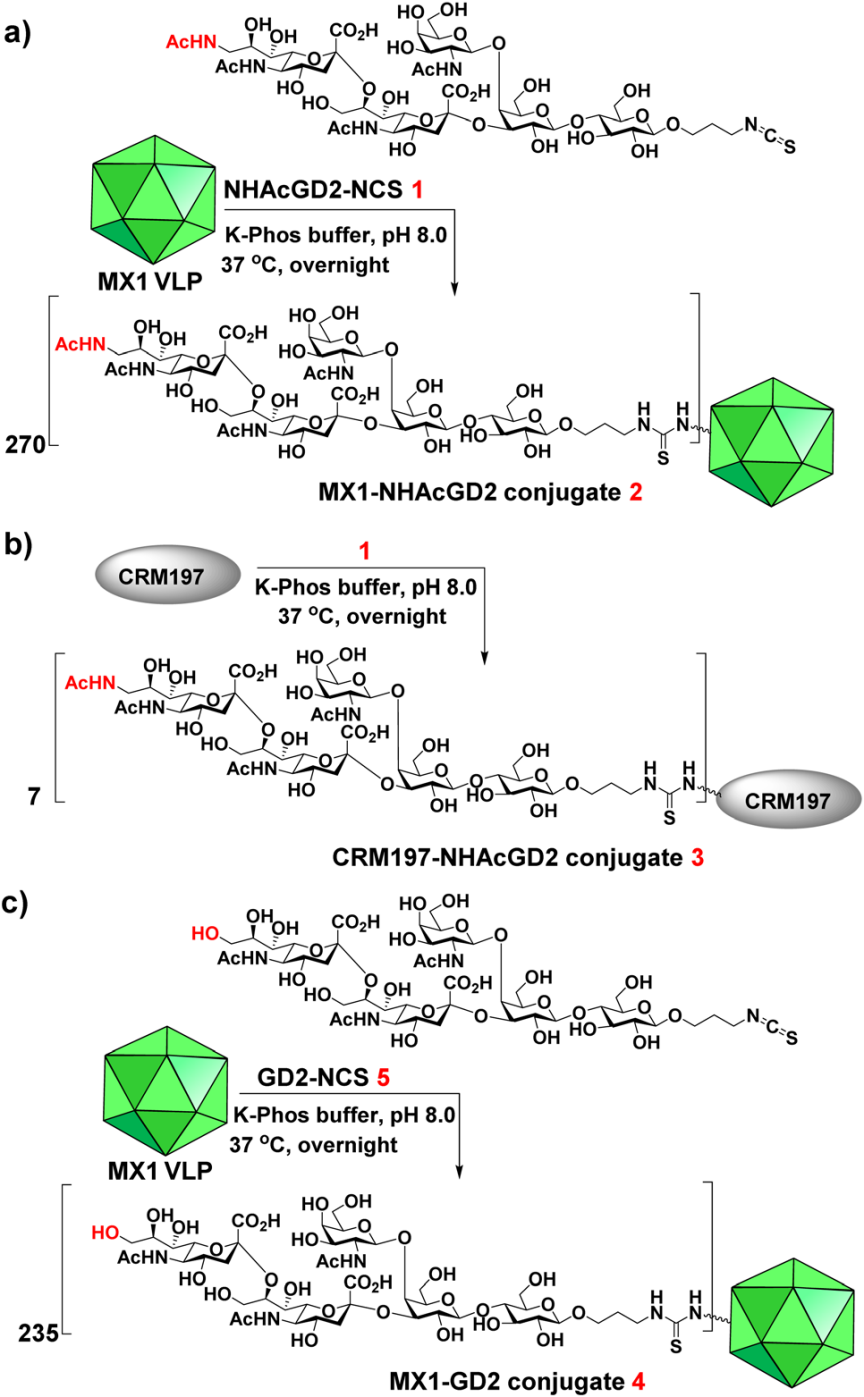
Syntheses of (a) MX1-NHAcGD2 conjugate 2, (b) CRM197-NHAcGD2 conjugate 3 and (c) MX1-GD2 conjugate 4.

We next exploited the MX1 carrier for the NHAcGD2 conjugate vaccine development. MX1-NHAcGD2 conjugate 2 was synthesized by adding 1 to K-Phos buffer (0.1 M, pH 8.0) with MX1 VLP overnight at 37 °C (Scheme 2a). MALDI-TOF MS analysis of the conjugate 2 showed that each MX1 particle contained an average of 270 NHAcGD2 (Fig. S5, ESI†). In parallel, the CRM197-NHAcGD2 conjugate 3 (Scheme 2b) was fabricated by coupling NHAcGD2-NCS 1 with cross-reactive material CRM197, which has been widely utilized as an anti-microbial vaccine and anticancer vaccine candidate.^44–46^ It was determined that 3 contained an average of 7 copies of NHAcGD2 per CRM197 (Fig. S6, ESI†). In addition, to verify the effect of 9-NHAc modification of GD2 on vaccine performance, MX1-GD2 conjugate 4 (Scheme 2c) was also made by conjugating GD2-NCS 5 (Scheme S2, ESI†) with MX1 VLP as a control vaccine, which contained 235 GD2 per particle (Fig. S7, ESI†).

### Immunological evaluation of MX1-NHAcGD2 conjugate 2, CRM197-NHAcGD2 conjugate 3 and MX1-GD2 conjugate 4

After synthesizing and characterizing conjugates 2–4, we assess their immune properties in C57BL/6 female mice. On days 0, 14, and 28, C57BL/6 female mice were vaccinated with conjugates 2–4 with monophosphoryl-lipid A (MPLA) as adjuvant. Pre- and post-immunization (day 35) sera were collected for immunological testing, including enzyme-linked immunosorbent assay (ELISA), fluorescence-activated cell sorting (FACS), and complement-dependent cytotoxicity (CDC) assay.

**Fig. 2:**
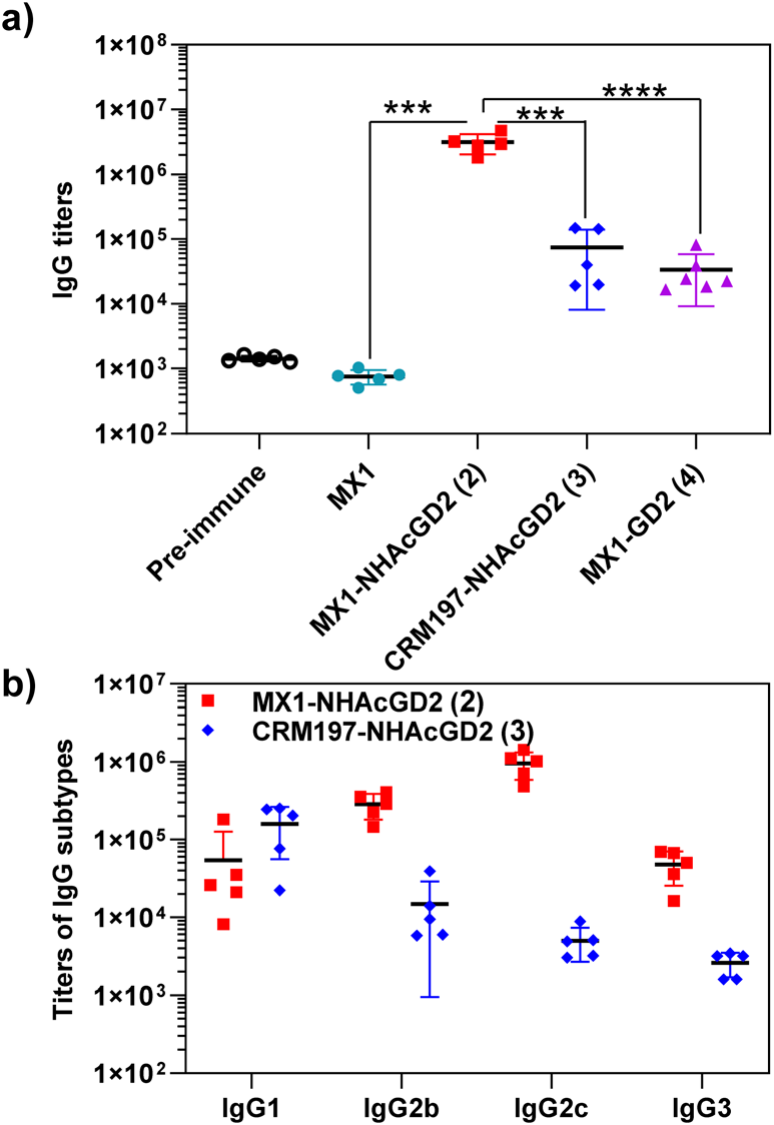
(a) Titers of anti-NHAcGD2 IgG antibodies elicited by MX1-NHAcGD2 (2) and CRM197-NHAcGD2 (3) in mice, as well as titers of anti-GD2 IgG antibodies elicited by MX1-GD2 (4). (b) IgG subtypes of sera from MX1-NHAcGD2 (2) and CRM197-NHAcGD2 (3) immunized mice. For detecting anti-NHAcGD2 IgG and anti-GD2 IgG titers, the ELISA measurements were performed against BSA-NHAcGD2 conjugate 6 and BSA-GD2 conjugate 7, respectively. Each symbol represents one mouse serum. The two-tailed unpaired Student's *t*-test of GraphPad Prism was used to determine the *p* values. ***p < 0.001, ****p < 0.0001.

Firstly, ELISA was used to detect anti-NHAcGD2/GD2 IgG antibody titers produced by the conjugates. To perform ELISA, NHAcGD2-NCS 1 and GD2-NCS 5 were respectively conjugated to bovine serum albumin (BSA) to give BSA-NHAcGD2 conjugate 6 and BSA-GD2 conjugate 7, which were characterized by MALDI-TOF MS (Fig. S8 and S9, ESI†), and then coated on plates for ELISA experiments. It was shown that pre-immunization, MX1-NHAcGD2 conjugate 2, and CRM197-NHAcGD2 conjugate 3-induced average anti-NHAcGD2 IgG titers were 1,425, 3 094 844, and 74 472, respectively (Fig. 2a), suggesting that MX1-NHAcGD2 conjugate 2 can elicit a more robust humoral immune response than CRM197-NHAcGD2 conjugate 3. Notably, the MX1-GD2 conjugate 4 induced an average anti-GD2 IgG titer of only 33 768 (Fig. 2a), which was significantly lower than the MX1-NHAcGD2 conjugate 2-induced IgG titer, indicating the importance of the 9NHAc modification of GD2 antigen. In addition, through the analysis of vaccine-induced IgG antibody subtypes, it was found that the IgG subtype produced by CRM197-NHAcGD2 conjugate 3 was predominantly IgG1. In contrast, the IgG produced by MX1-NHAcGD2 conjugate 2 had high levels of various subtypes, including IgG1, IgG2b, IgG2c, and IgG3, with IgG2c being the highest (Fig. 2b). Collectively, MX1-NHAcGD2 conjugate 2 produced high levels of 9-NHAc-GD2 specific IgG antibodies.

**Fig. 3:**
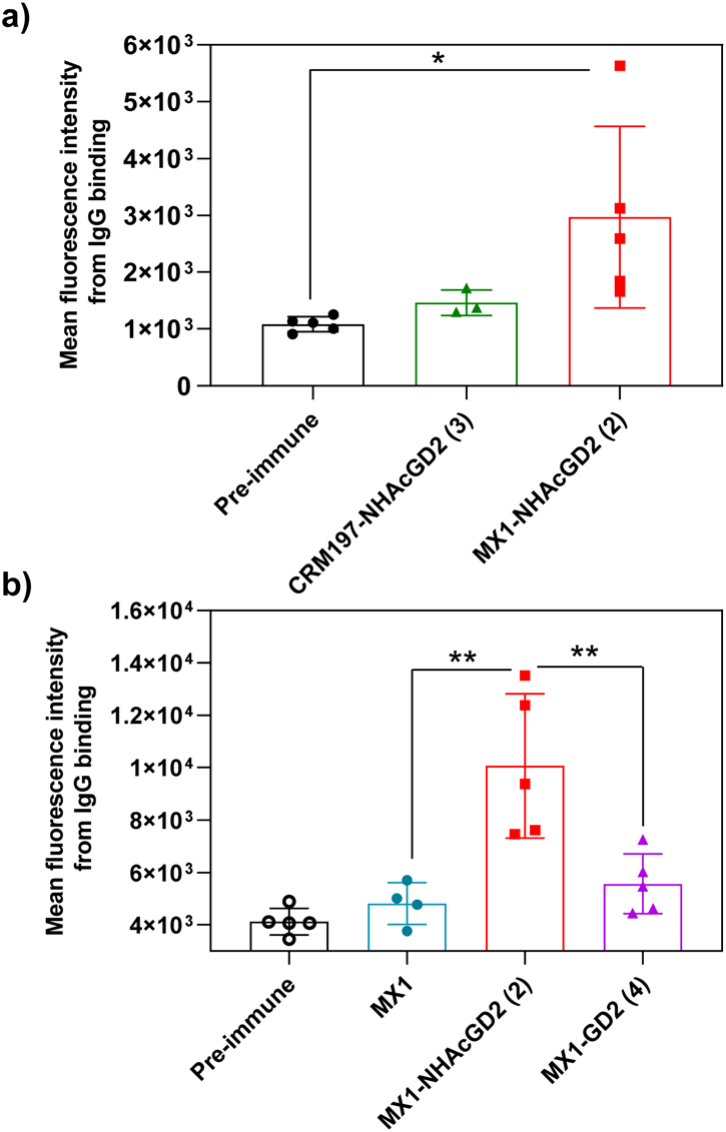
(a) Fluorescence-activated cell sorting (FACS) analysis showed that the binding ability of MX1-NHAcGD2 (2)-induced IgG to EL4 cells was superior to that of IgG produced by CRM197-NHAcGD2 (3) to EL4 cells. (b) FACS analysis showed that MX1-NHAcGD2 (2) elicited IgG with the strongest binding to IMR-32 cells compared with MX1-GD2 (4) and MX1-elicited antibodies. Each symbol represents a mouse. The assay was tested with a 1 : 20 dilution of serum. The two-tailed *t*-test of GraphPad Prism was used to determine the *p* values. *p < 0.05, **p < 0.01.

Secondly, FACS was used to detect the ability of serum antibodies to bind to tumors. The serum was co-incubated with tumor cells in FACS buffer. After washing the cells, the FITC-conjugated goat anti-mouse IgG antibody was added for flow cytometry analysis. GD2/9OAc-GD2 was expressed on the surface of EL4 lymphoma cells and human neuroblastoma IMR-32 cells, so these cells were used for FACS studies. The results showed MX1-NHAcGD2 conjugate 2-induced IgG antibody bound to EL4 cells more efficiently than CRM197-NHAcGD2 conjugate 3-generated IgG antibody (Fig. 3a). Notably, MX1 and MX1-GD2 (4) induced antibodies had a weaker binding capacity to IMR-32 cells. In contrast, the MX1-NHAcGD2 conjugate 2-induced IgG has the highest binding ability to IMR-32 cells (Fig. 3b). This result confirms that MX1-NHAcGD2 conjugate 2 produces potent IgG antibodies that bind tightly to tumor cells.

**Fig. 4:**
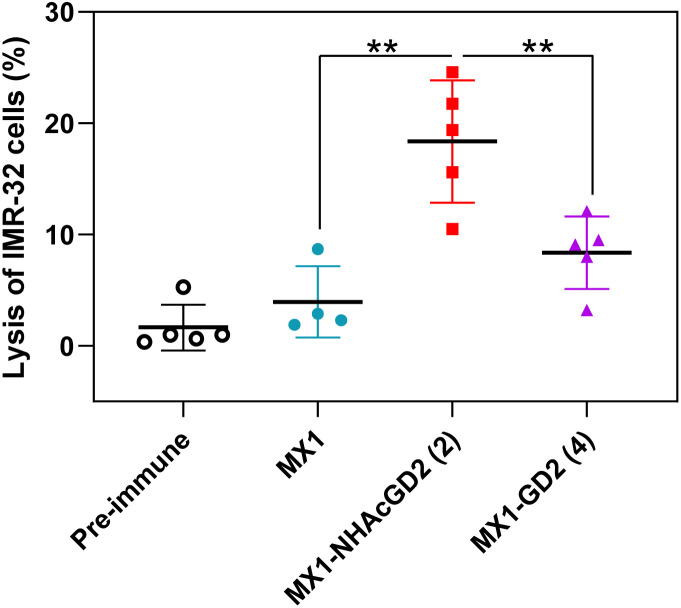
Sera from MX1-NHAcGD2 (2) exhibited significantly higher complement-dependent cytotoxicity (CDC) towards IMR-32 cells compared with those of MX1-GD2 (4) and MX1. CDC towards IMR-32 cells was determined by MTS assay. Each symbol represents a mouse (*n* = 4–5 mice for each group). The two-tailed *t*-test of GraphPad Prism was used to determine the *p* values. **p < 0.01.

Thirdly, the CDC was used to test the complement's ability to mediate cancer cell killing through serum antibodies. Complement cleaves target cells by binding specific antibodies to the corresponding antigen on the surface of tumor cells, activating the classical complement pathway known as the CDC. Post-immunization serum of MX1, MX1-NHAcGD2 conjugate 2, or MX1-GD2 conjugate 4 was incubated with IMR-32 cells, rabbit complement was then added, and cytotoxicity was calculated using the MTS cell viability assay. The results showed that the proportion of IMR-32 cells lysed upon incubation with MX1-NHAcGD2 conjugate 2 induced IgG was significantly higher than those treated with MX1-GD2 conjugate 4 and MX1-induced IgG (Fig. 4), indicating that the IgG produced by the conjugate 2 had a superior killing effect on IMR-32 cells in the presence of complement.

### MX1 VLP is also a superior carrier for NHAcGD3 conjugate vaccine development

**Scheme 3:**
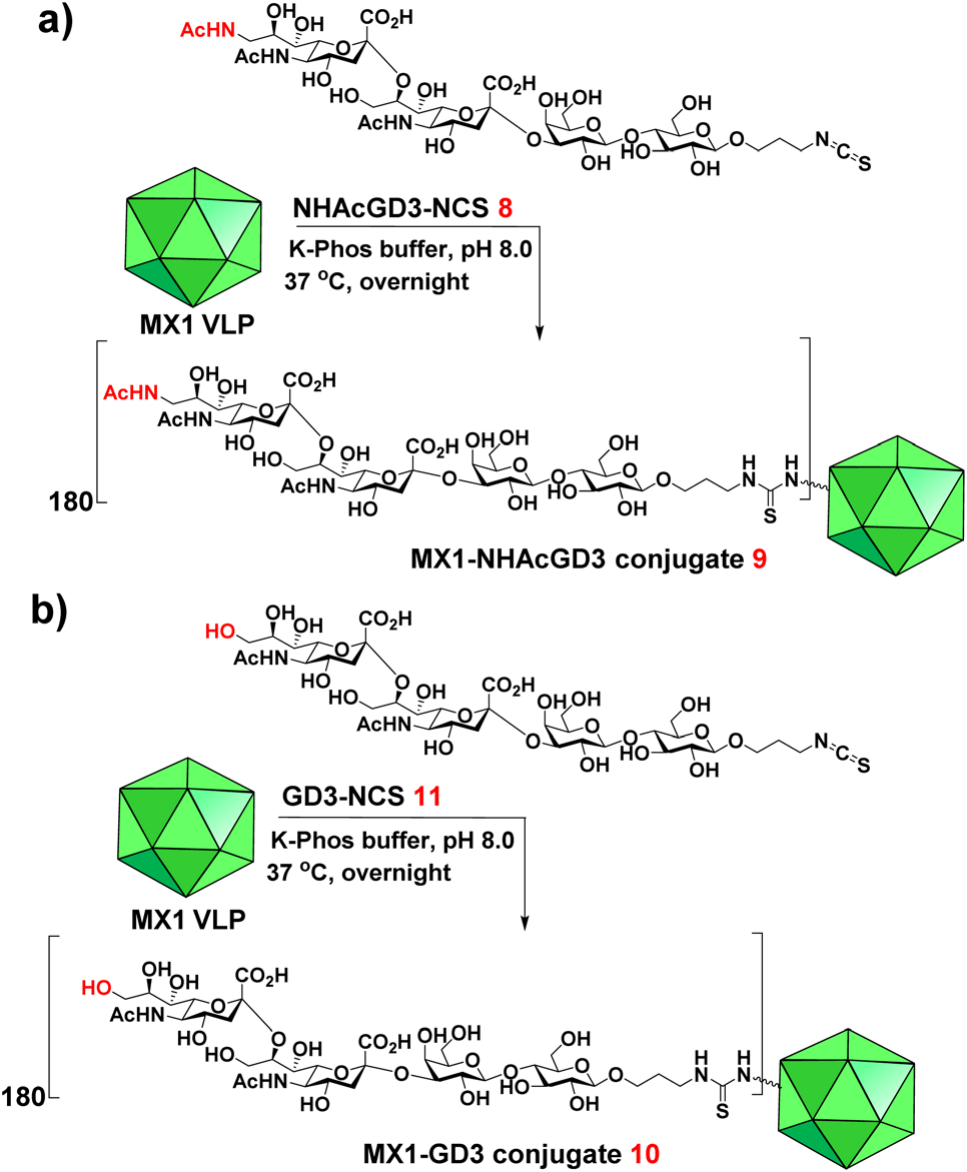
Syntheses of (a) MX1-NHAcGD3 (9) and (b) MX1-GD3 (10).

9-OAc-GD3 is another important ganglioside antigen that is overexpressed in melanoma,^23^ glioblastoma,^24^ breast cancer,^25^ and small cell lung cancer.^26^ Inspired by the superiority of the NHAcGD2 antigen, we envision NHAcGD3 as a promising antigen that mimics 9OAc-GD3. To synthesize the 9-NHAc-GD3 antigen for MX1 conjugation, we converted the amine group of NHAcGD3-NH2 S10 to NHAcGD3-NCS 8 with thiophosgene (Scheme S3a, ESI†). 8 was then conjugated with the bacteriophage MX1 VLP in K-Phos buffer (0.1 M, pH 8.0) overnight at 37 °C to give MX1-NHAcGD3 conjugate 9 (Scheme 3a). MALDI-TOF MS analysis of the resulting conjugate showed that the number of NHAcGD3 per MX1 particle was 180 (Fig. S10, ESI†). To benchmark the performance of the 9NHAc modification, we also synthesized MX1-GD3 conjugate 10(Scheme 3b) by conjugating MX1 VLP with GD3-NCS 11 (Scheme S3b, ESI†) in K-Phos buffer (pH 8.0, 0.1 M). MALDI-TOF MS determined the amount of GD3 in MX1-GD3 (10) as 180 copies of GD3 per particle (Fig. S11, ESI†).

**Fig. 5:**
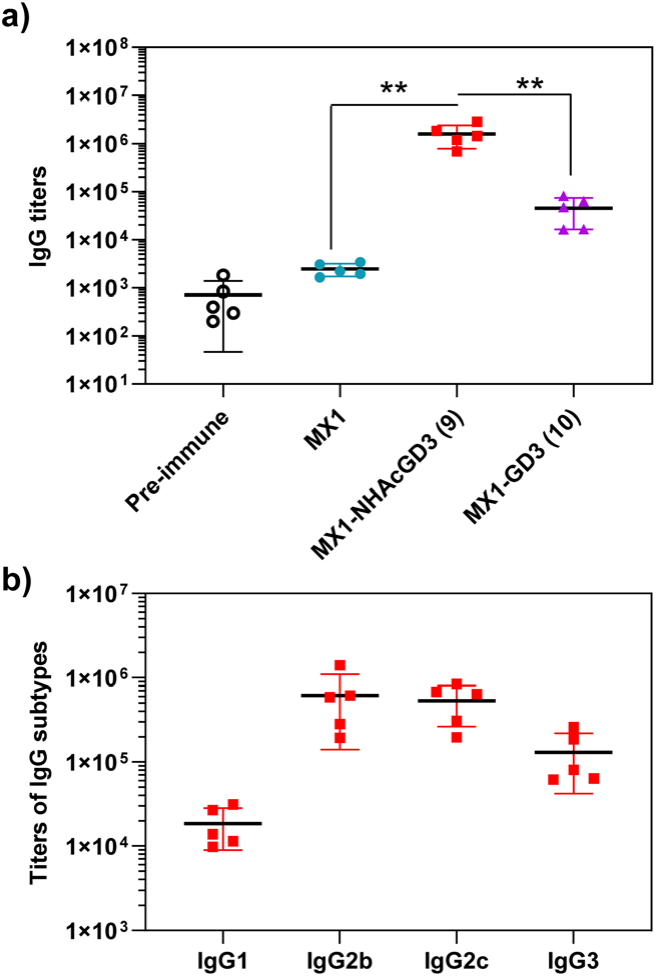
(a) Titers of anti-NHAcGD3 IgG antibodies elicited by MX1-NHAcGD3 (9), and titers of anti-GD3 IgG antibodies elicited by MX1-GD3 (10). (b) IgG subtypes of mice immunized with MX1-NHAcGD3 (9). For testing levels of anti-NHAcGD3 IgG and anti-GD3 IgG, the ELISA measurements were performed against BSA-NHAcGD3 (12) and BSA-GD3 (13), respectively. The two-tailed *t*-test of GraphPad Prism was used to determine the *p* values. **p < 0.01.

With conjugate vaccines 9 and 10 in hand, we measured the anti-NHAcGD3/GD3 IgG titers they produced in C57BL/6 mice. NHAcGD3-NCS 8 and GD3-NCS 11 were conjugated to BSA to obtain BSA-NHAcGD3 conjugate 12 and BSA-GD3 conjugate 13, which were characterized with MALDI-TOF MS (Fig. S12 and S13, ESI†). When ELISA was performed, the levels of anti-NHAcGD3 IgG and anti-GD3 IgG were against 12 and 13, respectively. The results showed that the mean anti-NHAcGD3 IgG titers by pre-immunization and those induced by MX1, and MX1-NHAcGD3 conjugate 9 were 711, 1626, 1 603 672, respectively (Fig. 5a). Notably, the mean anti-GD3 IgG titer caused by MX1-GD3 conjugate 10 was only 45 054 (Fig. 5a), which was significantly lower than that induced by MX1-NHAcGD3 conjugate 9, indicating the crucial role of 9NHAc modification of GD3 antigen. The vaccine-induced IgG antibody subtype analysis revealed that MX1-NHAcGD3 conjugate 9 produced high levels of various subtypes of IgG, with IgG2b and IgG2c predominating (Fig. 5b). Overall, MX1-NHAcGD3 conjugate 9 induces a high NHAcGD3-specific IgG response.

**Fig. 6:**
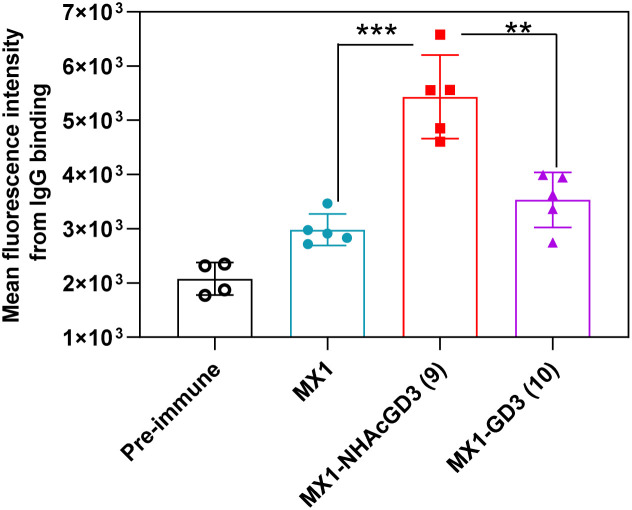
FACS analysis showed MX1-NHAcGD3 (9)-induced IgG antibody bound to SK-MEL-28 more efficiently than MX1-GD3 (10)-generated IgG antibody. The assay was tested with 1 : 20 dilution of the corresponding sera. The two-tailed *t*-test of GraphPad Prism was used to determine the *p* values. **p < 0.01, ***p < 0.001.

Next, FACS was used to test the ability of antibodies produced by GD3-related conjugate vaccines to bind to tumors. GD3/9OAc-GD3 was expressed on the surface of human melanoma SK-MEL-28 cell line, which was used in FACS studies. The results showed that MX1 and MX1-GD3 conjugate 10-induced IgG antibodies had a weak binding capacity to SK-MEL-28 cells. In contrast, MX1-NHAcGD3 conjugate 9 induced-IgG had strong binding to SK-MEL-28 cells (Fig. 6). This result confirms that MX1-NHAcGD3 conjugate 9 produces potent NHAcGD3-specific IgG antibodies that bind tightly to tumor cells.

**Fig. 7:**
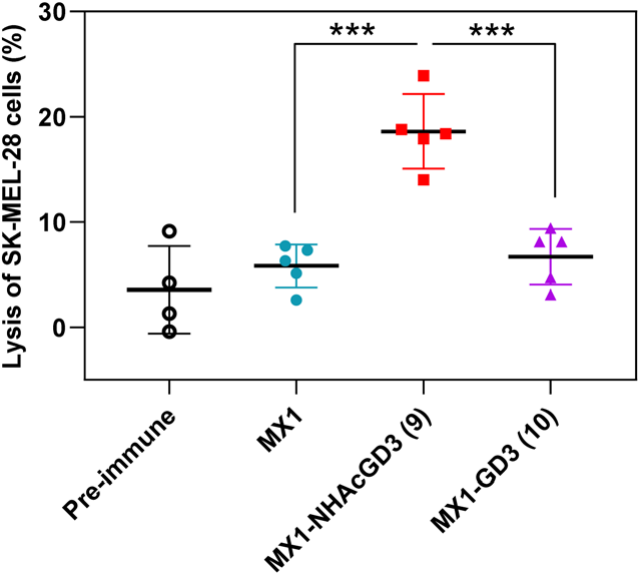
Sera from MX1-NHAcGD3 (9) vaccination exhibited significantly higher complement-dependent cytotoxicity (CDC) towards SK-MEL-28 cells compared with those from MX1 and MX1-GD3 (10) vaccination. Each symbol represents a mouse (*n* = 4–5 mice for each group). The *p* values were determined through a two tailed *t* test using GraphPad Prism. ***p < 0.001.

Finally, post-immunization serum of MX1, MX1-NHAcGD3 conjugate 9, or MX1-GD3 conjugate 10 was incubated with SK-MEL-28 cells, rabbit complement was added, and cytotoxicity was calculated using the MTS assay. The results showed that the lysis proportion of SK-MEL-28 cells treated with MX1-NHAcGD3 conjugate 9 induced IgG antibodies was significantly higher than those treated with MX1-GD3 conjugate 10 or MX1-induced IgG antibodies (Fig. 7), suggesting that the IgG produced by the conjugate 9 had a superior killing effect on SK-MEL-28 cells in the presence of complement.

## Conclusions

In this work, a novel bacteriophage MX1 VLP is reported to construct the MX1-NHAcGD2 conjugate by conjugating the 9NHAc-GD2 (NHAcGD2) antigen with MX1 VLP. The MX1-NHAcGD2 conjugate produces potent NHAcGD2-specific IgG antibodies that bind specifically to IMR-32 neuroblastoma cells, and antibodies mediate good CDC to kill this cell line. In addition, inspired by the NHAcGD2 antigen, the 9NHAc-GD3 (NHAcGD3) antigen used to mimic the 9-OAc-GD3 antigen is investigated for the first time. The conjugate of MX1 to NHAcGD3 induces potent NHAcGD3-specific IgG antibodies, which bound firmly to SK-MEL-28 melanoma cells and mediated significant CDC killing. In summary, the MX1 VLP has excellent potential as a new class of VLP vaccine carrier. The MX1-NHAcGD2 and MX1-NHAcGD3 conjugates can be exciting leads for anticancer vaccines.

## Author contributions

X. W. conceived the concept of developing MX1-NHAcGD2 and MX1-NHAcGD3 conjugate vaccines and supervised the project. X. W. and Q. Z. contributed to experimental design. Q. Z. performed the experiments. X. W. and Q. Z. wrote the manuscript. All authors revised the manuscript.

## Conflicts of interest

The authors declare no conflict of interest.

## Supplementary Material

RA-014-D3RA08923A-s001

RA-014-D3RA08923A-s002

RA-014-D3RA08923A-s003
