# Supplementary material for: Development of NHAcGD2/NHAcGD3 conjugates of bacteriophage MX1 virus-like particles as anticancer vaccines
Source: RSC Adv. 2024 Feb 19;14(9):6246–52. doi: 10.1039/d3ra08923a (PMC10875654; doi:10.1039/d3ra08923a)
Supplement: RA-014-D3RA08923A-s001 [file RA-014-D3RA08923A-s001.pdf]

3.86 – 3.50 (m, 19H), 3.46 (dd, J = 9.2, 1.9 Hz, 1H), 3.36 – 3.25 (m, 2H), 3.15 (t, J = 7.2 Hz, 2H), 2.76 (dd, J = 12.4, 4.6 Hz, 1H), 2.66 (dd, J = 12.3, 4.4 Hz, 1H), 2.04 (s, 3H), 2.02 (s, 3H), 2.01 (s, 3H), 1.73 (td, J = 12.2, 6.4 Hz, 2H); ^{13}C NMR (150 MHz, D_2O) δ 174.85, 174.46, 173.27, 173.23, 102.64, 102.04, 100.51, 98.00, 78.08, 77.91, 75.40, 75.17, 74.72, 74.14, 74.00, 72.69, 72.40, 69.75, 69.56, 69.18, 69.09, 68.37, 67.82, 67.79, 67.45, 65.85, 61.42, 61.01, 59.81, 52.16, 51.61, 42.19, 40.38, 39.49, 37.51, 26.58, 22.19, 21.93, 21.73. HRMS (ESI) m/z calcd for $C_{39}H_{65}N_4O_{27}$ $[M-H]^-$ 1021.3842, found 1021.3723.

NHAcGD3-NCS 8

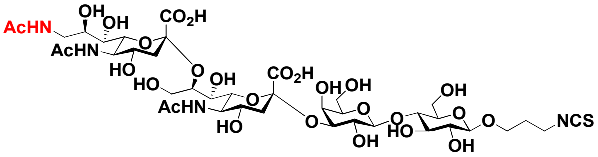

8

S10 (15 mg, 0.015 mmol) was dissolved in an aqueous $NaHCO_3$ solution (500 µL, 10 mg/mL). To the solution was added chloroform (750 µL) containing thiophosgene (1.67 µL, 21.8 µmol). The reaction solution was stirred at RT for 3 h. The reaction mixture was then diluted with water. The aqueous layer was extracted twice with chloroform and freeze-dried to give NHAcGD3-NCS **8**. ^1H NMR (600 MHz, D_2O) δ 4.31 – 4.20 (m, 2H), 3.96 – 3.82 (m, 3H), 3.81 – 3.21 (m, 27H), 3.16 – 2.99 (m, 2H), 2.51 (dd, J = 12.5, 4.5 Hz, 1H), 2.41 (dd, J = 12.3, 4.3 Hz, 1H), 1.80 (d, J = 1.6 Hz, 3H), 1.78 (s, 3H), 1.77 (s, 3H), 1.49 (q, J = 11.7 Hz, 2H). HRMS (ESI) m/z calcd for $C_{40}H_{63}N_4O_{27}S$ $[M-H]^-$ 1063.3406, found 1063.3273.

GD3-NH$_2$ S11

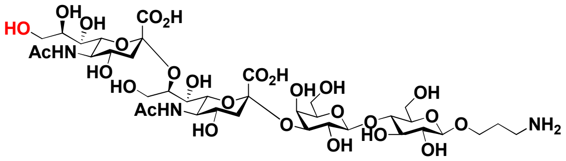

S11

S7 (20 mg, 0.02 mmol) was dissolved in H_2O/MeOH (2 mL, 1:1 v/v). To this solution was added Pd/C (10 wt.% loading, 10 mg). The atmosphere was removed by vacuum and replaced by H_2, and the reaction solution was stirred under H_2 for 12 h.
