# Supplementary material for: Development of NHAcGD2/NHAcGD3 conjugates of bacteriophage MX1 virus-like particles as anticancer vaccines
Source: RSC Adv. 2024 Feb 19;14(9):6246–52. doi: 10.1039/d3ra08923a (PMC10875654; doi:10.1039/d3ra08923a)
Supplement: RA-014-D3RA08923A-s002 [file RA-014-D3RA08923A-s002.pdf]

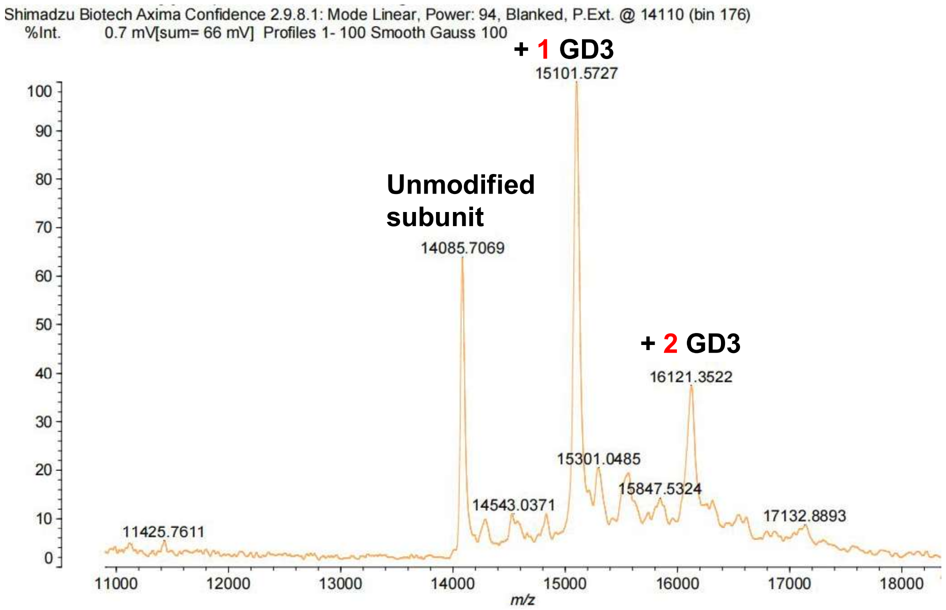

Fig. S11 MALDI-TOF MS for MX1-GD3 conjugate **10**. MS analysis of MX1-NHAcGD3 conjugate **10** showed an average of 180 copies of GD3 on each MX1 particle.

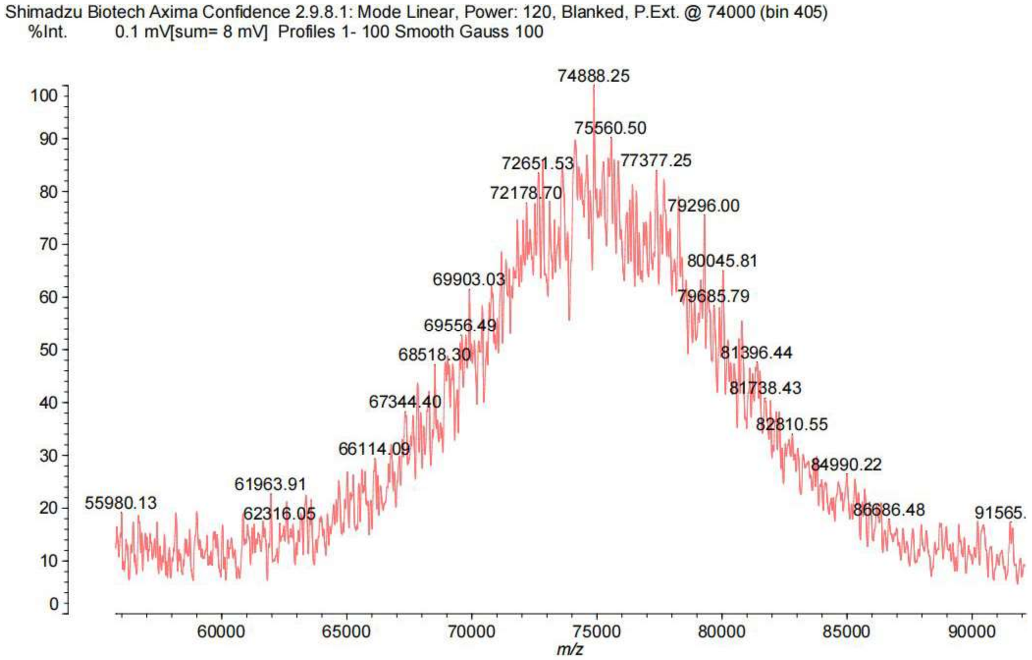

Fig. S12 MALDI-TOF MS characterization of BSA-NHAcGD3 conjugate **12** for ELISA. MS analysis of the conjugate **12** showed that the number of NHAcGD3 per BSA was 8 on average.
