# Supplementary material for: Development of NHAcGD2/NHAcGD3 conjugates of bacteriophage MX1 virus-like particles as anticancer vaccines
Source: RSC Adv. 2024 Feb 19;14(9):6246–52. doi: 10.1039/d3ra08923a (PMC10875654; doi:10.1039/d3ra08923a)
Supplement: RA-014-D3RA08923A-s003 [file RA-014-D3RA08923A-s003.pdf]

Electronic Supplementary Material (ESI) for RSC Advances.
This journal is © The Royal Society of Chemistry 2024

Supporting information

Development of NHAcGD2/NHAcGD3 conjugates of bacteriophage MX1 virus-like particles as anticancer vaccines

Qingyu Zhao,[a] Xuefei Huang,[b] and Xuanjun Wu[a,*]

[a]National Glycoengineering Research Center, and Shandong Key Laboratory of Carbohydrate Chemistry and Glycobiology, NMPA Key Laboratory for Quality Research and Evaluation of Carbohydrate-based Medicine, Shandong University, Qingdao, Shandong 266237, China

[b]Departments of Chemistry and Biomedical Engineering, Institute for Quantitative Health Science and Engineering, Michigan State University, East Lansing, Michigan 48824, United States

Email: xuanjun@sdu.edu.cn

Table of Contents

Fig. S1 Size-exclusion HPLC characterization of MX1 VLP. **S3**

Fig. S2 SDS polyacrylamide gel electrophoresis (SDS-PAGE) characterization of MX1 VLP. **S3**

Fig. S3 MALDI-TOF mass spectrometry (MS) characterization of MX1 capsid subunit. **S3**

Fig. S4 Dynamic light scattering (DLS) analysis of MX1 VLP. **S4**

Fig. S5 MALDI-TOF MS for MX1-NHAcGD2 conjugate **2**. **S4**

Fig. S6 MALDI-TOF MS results for (a) CRM197 and (b) CRM197-NHAcGD2 conjugate **3**. **S5**

Fig. S7 MALDI-TOF MS for MX1-GD2 conjugate **4**. **S6**

Fig. S8 MALDI-TOF MS characterization of BSA-NHAcGD2 conjugate **6** for ELISA. **S6**

Fig. S9 MALDI-TOF MS characterization of BSA-GD2 conjugate **7** for ELISA. **S7**

Fig. S10 MALDI-TOF MS for MX1-NHAcGD3 conjugate **9**. **S7**

Fig. S11 MALDI-TOF MS for MX1-GD3 conjugate **10**. **S8**

Fig. S12 MALDI-TOF MS characterization of BSA-NHAcGD3 conjugate **12** for ELISA. **S8**

Fig. S13 MALDI-TOF MS characterization of BSA-GD3 conjugate **13** for ELISA. **S9**

General experimental procedures and methods for synthesis S9

Expression and purification of MX1 VLP S10

Synthesis of NHAcGD2-isothiocyanate (NCS) 1 S12

Synthesis of GD2-NCS 5 S17

Synthesis of MX1-NHAcGD2 conjugate 2 and MX1-GD2 conjugate 4 S22

Synthesis of CRM197-NHAcGD2 conjugate 3 S23

Synthesis of MX1-NHAcGD3 conjugate 9 and MX1-GD3 conjugate 10 S23

Synthesis of BSA-NHAcGD2 conjugate 6, BSA-GD2 conjugate 7, BSA-NHAcGD3 conjugate 12 and BSA-GD3 conjugate 13 S23

Mouse immunization S24

Enzyme-linked immunosorbent assay (ELISA) S24

Fluorescence-activated cell sorting (FACS) S24

Complement-dependent cytotoxicity (CDC) assay S25

Product Characterization Spectra S26-S41

References S41

Supporting Figures

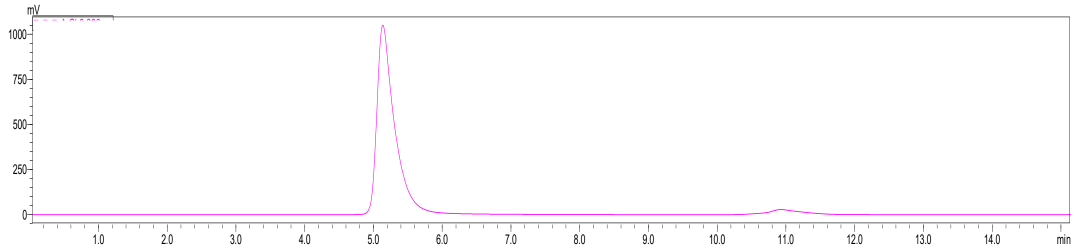

Fig. S1 Size-exclusion HPLC characterization of MX1 VLP.

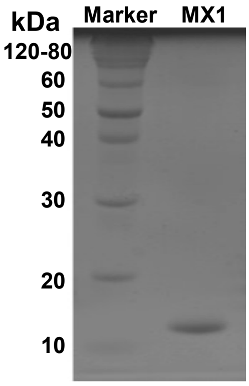

Fig. S2 SDS polyacrylamide gel electrophoresis (SDS-PAGE) characterization of MX1 capsid subunit.

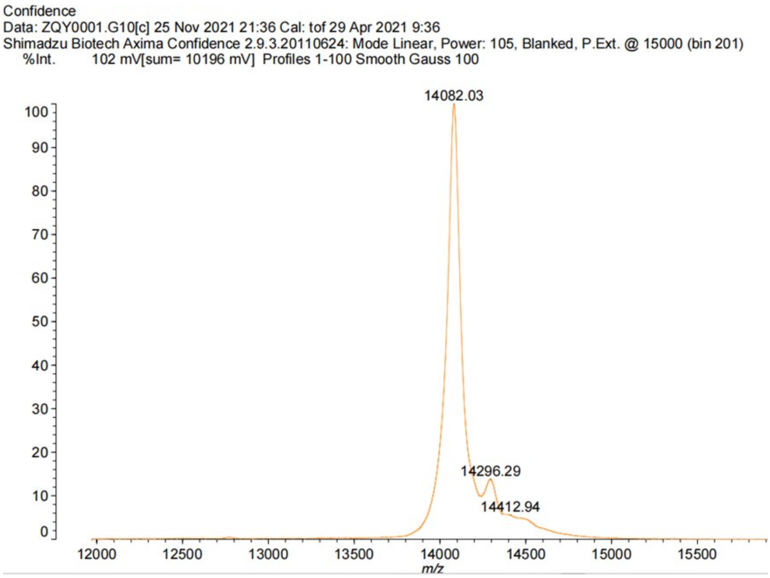

Fig. S3 MALDI-TOF mass spectrometry (MS) characterization of MX1 capsid subunit. The result showed that the MX1 capsid subunit had a molecular weight of 14.1 kDa.

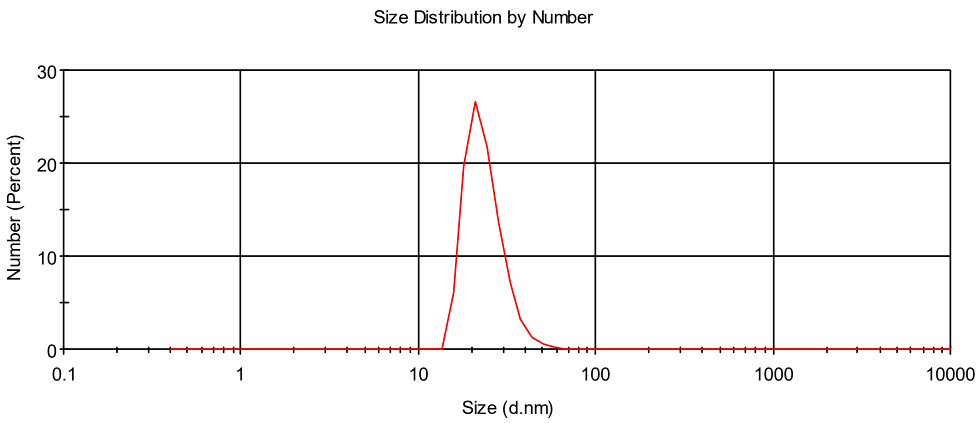

Fig. S4 Dynamic light scattering (DLS) analysis of MX1 VLP. The result showed that the average diameter of MX1 VLP in K-Phos buffer (pH = 7.4) was 29 nm.

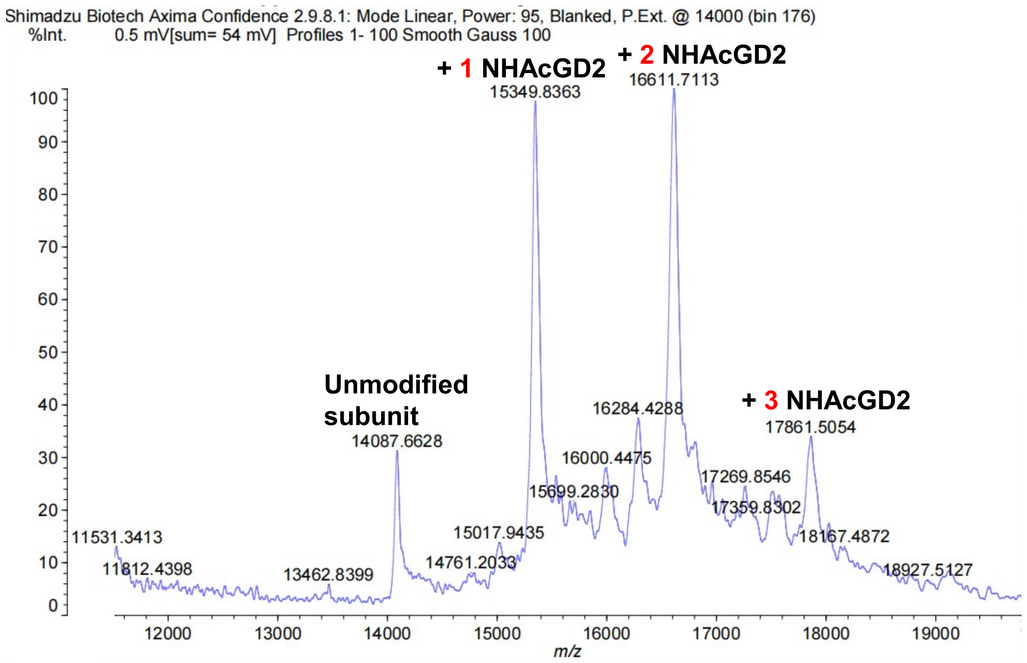

Fig. S5 MALDI-TOF MS for MX1-NHAcGD2 conjugate **2**. MS analysis of MX1-NHAcGD2 conjugate **2** revealed an average of 270 copies of NHAcGD2 per MX1 particle.

a) CRM197

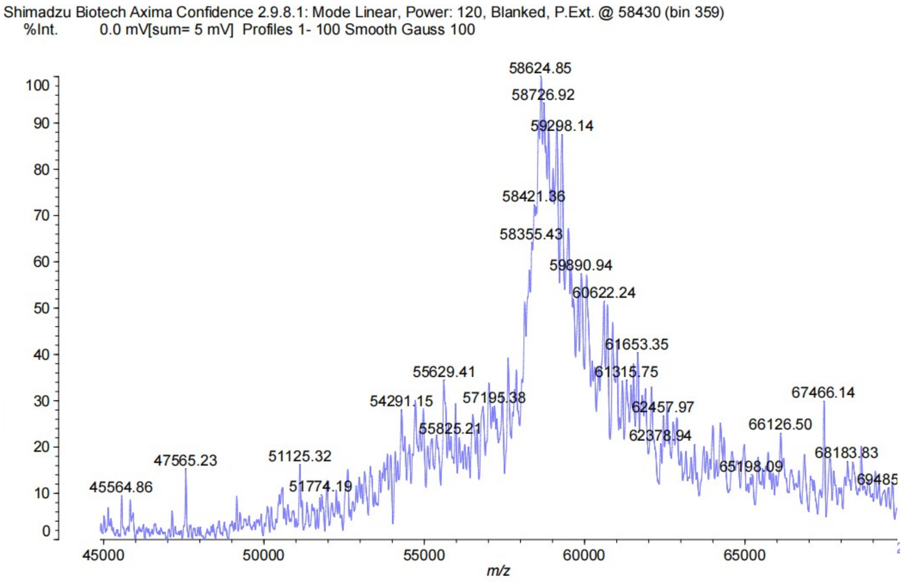

b) CRM197-NHAcGD2 conjugate 3

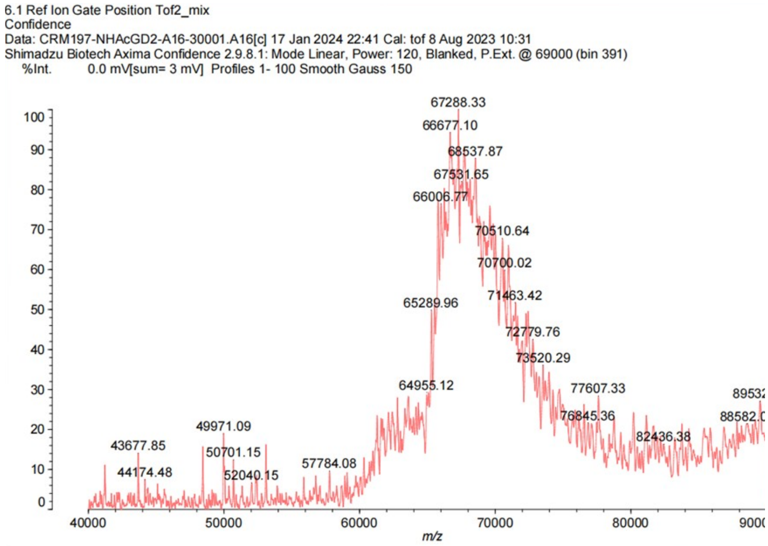

Fig. S6 MALDI-TOF MS results for (a) CRM197 and (b) CRM197-NHAcGD2 conjugate **3**. The molecular weight of CRM197 shifted from 58.6 kDa to 67.3 kDa after conjugation. The difference of MW before and after conjugation divided by the MW of NHAcGD2 (1268) gave the average loading of 7 copies of NHAcGD2 per CRM197.

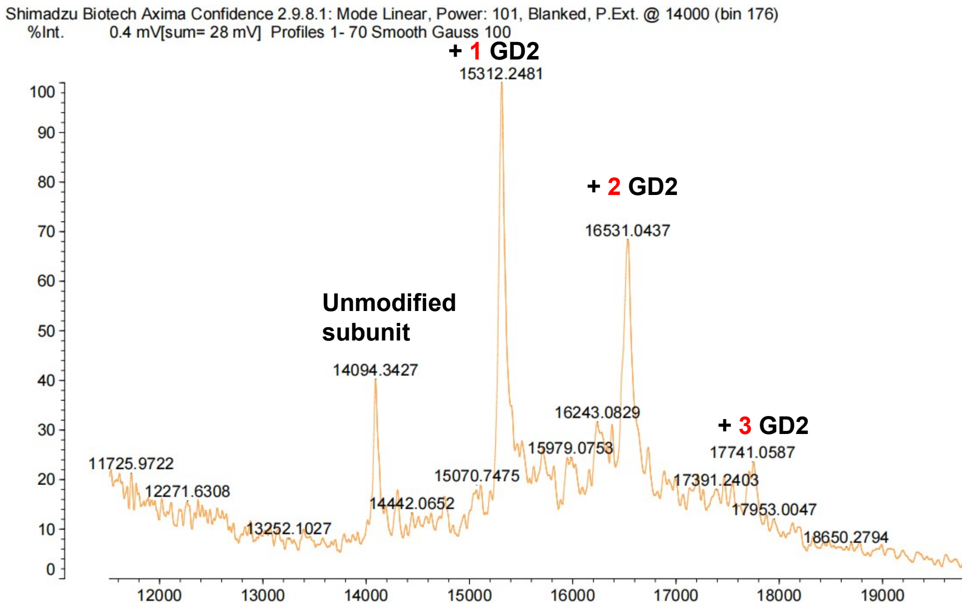

Fig. S7 MALDI-TOF MS for MX1-GD2 conjugate **4**. MS analysis of MX1-GD2 conjugate **4** showed an average of 235 copies of GD2 on each MX1 particle.

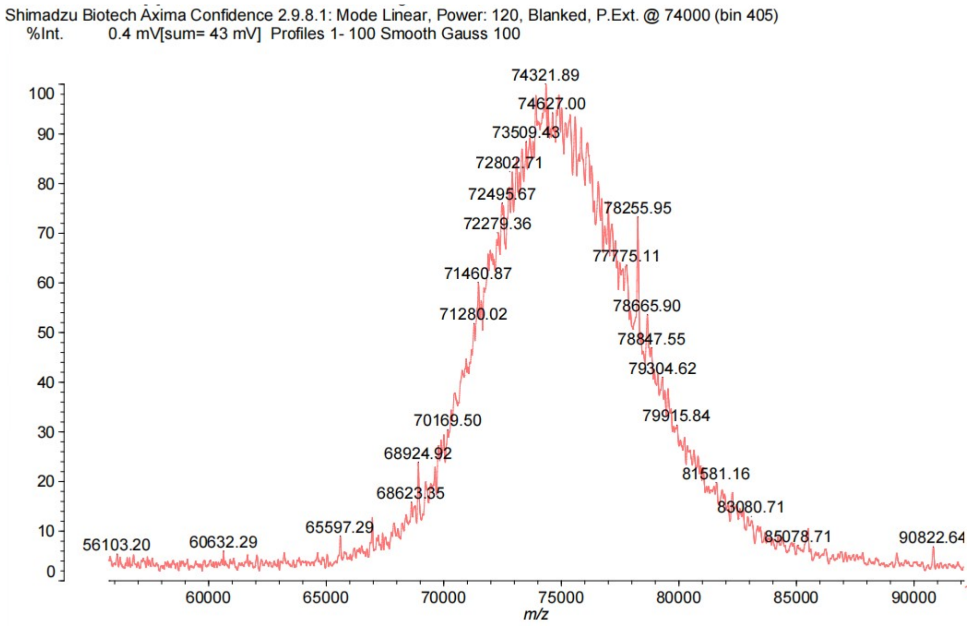

Fig. S8 MALDI-TOF MS characterization of BSA-NHAcGD2 conjugate **6** for ELISA. The molecular weight shifted from 66.4 kDa of BSA to 74.3 kDa of the conjugate **6**. The difference of MW before and after conjugation divided by the MW of NHAcGD2 (1268) gave the average loading of 6 copies of NHAcGD2 per BSA molecule.

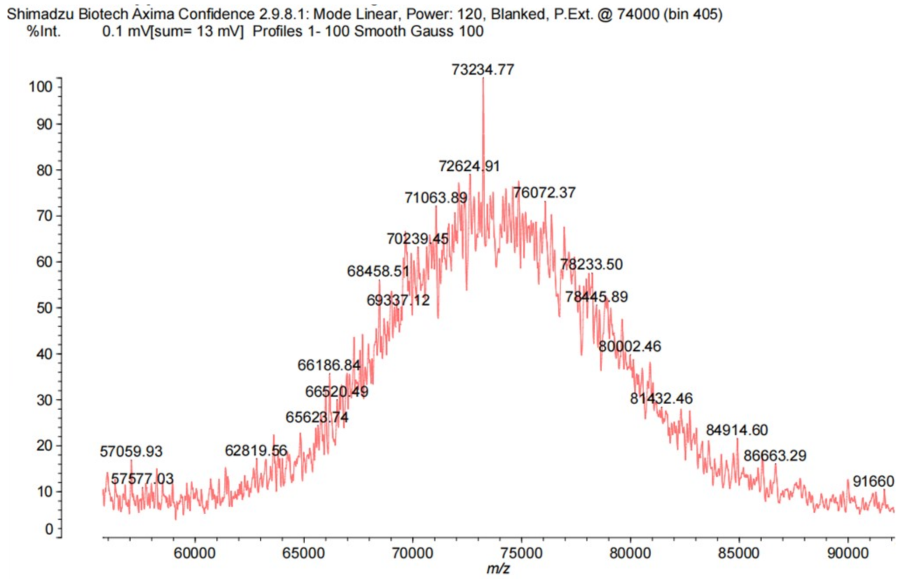

Fig. S9 MALDI-TOF MS characterization of BSA-GD2 conjugate **7** for ELISA. MS analysis of the BSA-GD2 conjugate **7** showed that the number of GD2 per BSA was 5 on average.

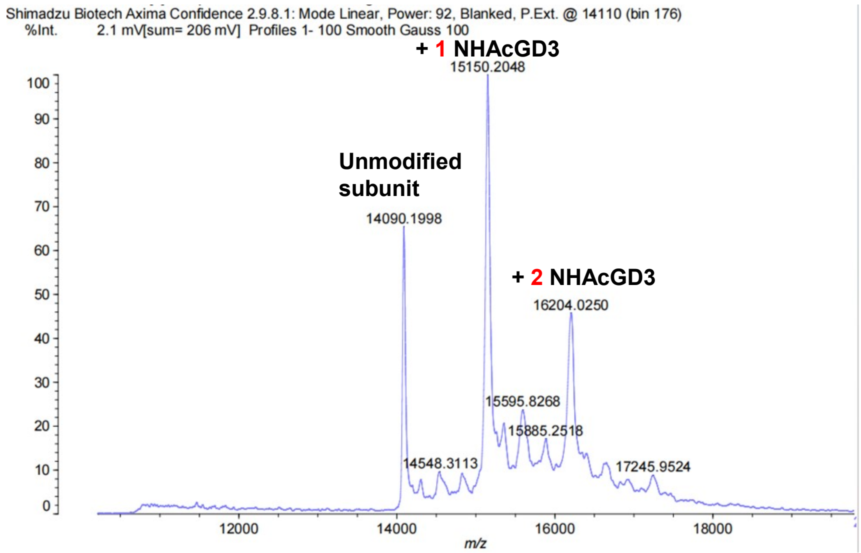

Fig. S10 MALDI-TOF MS for MX1-NHAcGD3 conjugate **9**. MS analysis of MX1-NHAcGD3 conjugate **9** showed an average loading of 180 copies of NHAcGD3 per MX1 particle. It should be noted that the coupling efficiency of NHAcGD3 to MX1 is slightly lower than that of NHAcGD2 to MX1, which may be due to the slightly different stability of the NCS group between NHAcGD2-NCS and NHAcGD3-NCS in K-Phos buffer (pH 8). In future studies, the number of NHAcGD3 on MX1 can be increased by adding more NHAcGD3-NCS for MX1 conjugation.

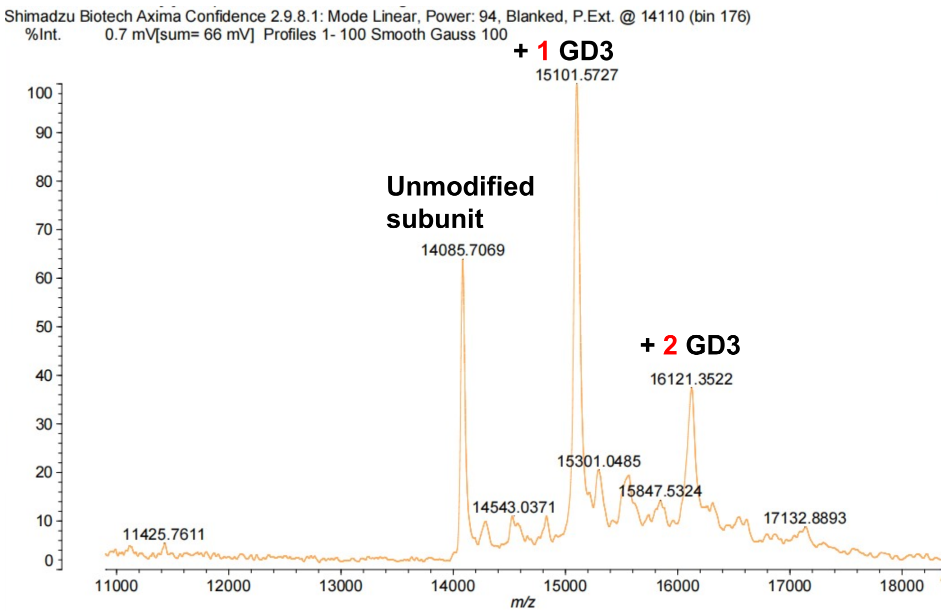

Fig. S11 MALDI-TOF MS for MX1-GD3 conjugate **10**. MS analysis of MX1-NHAcGD3 conjugate **10** showed an average of 180 copies of GD3 on each MX1 particle.

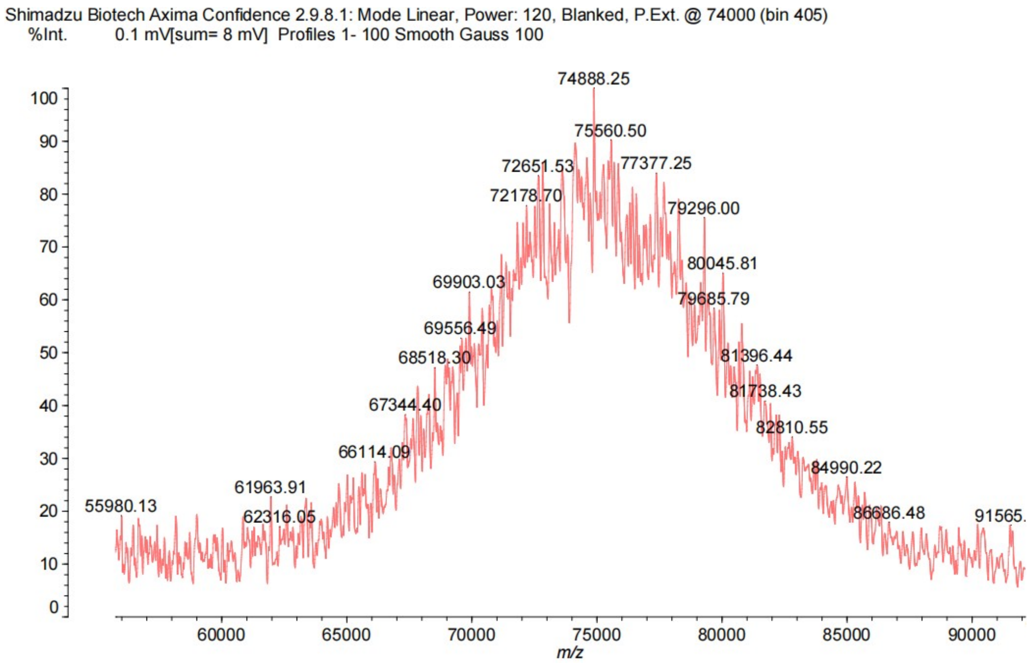

Fig. S12 MALDI-TOF MS characterization of BSA-NHAcGD3 conjugate **12** for ELISA. MS analysis of the conjugate **12** showed that the number of NHAcGD3 per BSA was 8 on average.

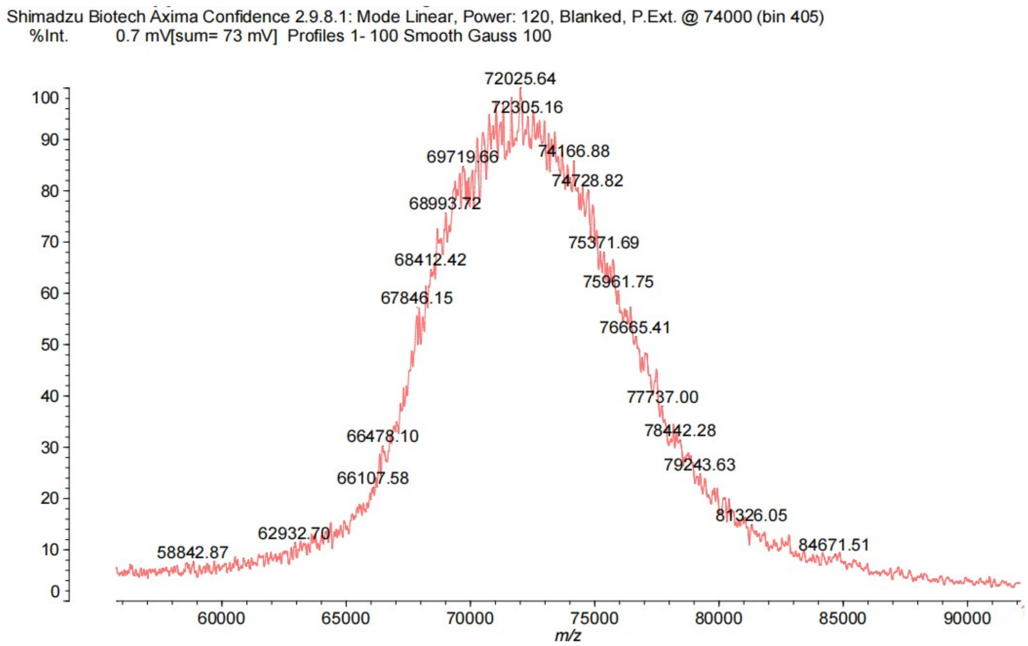

Fig. S13 MALDI-TOF MS characterization of BSA-GD3 conjugate **13** for ELISA. MALDI-TOF MS of BSA-GD3 conjugate **13**. MS analysis of the conjugate **13** showed that the number of GD3 per BSA was 5 on average.

General experimental procedures and methods for synthesis

All chemicals were reagent grade and received from the manufacturer unless otherwise noted. Thiophosgene was purchased from Shanghai Macklin. Pd/C were purchased from J&K Scientific Ltd. HRP-conjugated goat anti-mouse IgG, IgG1, IgG2b, IgG2c, and IgG3 were all derived from Abcam. FITC Goat anti-mouse IgG (minimal x-reactivity) antibody was purchased from BioLegend. The CellTiter 96® AQ$_{\text{ueous}}$ One Solution Reagent was purchased from Promega. Gel filtration chromatography was performed using a column (100 cm × 2.5 cm) filled with the BioGel P-2 Fine resin (Bio-Rad). Ion exchange chromatography was performed using a column (100 cm × 2.5 cm) packed with DEAE Sepharose Fast Flow resin (Cytiva). Centrifugal filter units of 100 KDa molecular weight cut-off (MWCO) were purchased from EMD Millipore. NMR spectra were calibrated using solvent signals (^{1}H: δ 4.79 for D_2O).

CMP-sialic acid synthetase (*Neisseria meningitidis*, NmCSS),[1] α2,3-sialyltransferase (*Pasteurella multocida*, PmST1),[2] α2,8-sialyltransferase

(*Campylobacter jejuni*, CjCstII),[3] β1,4-*N*-acetylgalactosaminyltransferase (*Campylobacter jejuni*, CjCgtA),[4] were expressed and purified as reported.

MALDI-TOF MS analysis was performed for characterization of the conjugates. Samples for MALDI-TOF were prepared as follows: the conjugate (**2−4**, **6**, **7**, **9**, **10**, **12**, or **13**, 10 mg/mL) was mixed with 100 mM DTT (1:1, v/v) and then incubated in a water bath at 37 °C for 30 min. After desalting using Cleanup C18 Pipette Tips (Agilent Technologies), the sample (2 μL) and matrix solution (2 μL, 10 mg/mL sinapic acid in 50:50:0.1 CH_3CN/H_2O/TFA) were mixed and spotted on a MALDI plate, air-dried (3 rounds) and then analyzed by MALDI-TOF MS.

IMR-32 and SK-MEL-28 cells were obtained from the American Culture Collection (ATCC) and cultured in Minimum Essential Medium (MEM) containing 10% fetal bovine serum (FBS), penicillin (100 U/mL) and streptomycin (100 μg/mL) at 37 °C in a 5% CO_2 incubator. C57BL/6 female mice aged 6−8 weeks were provided from the Laboratory Animal Center of Shandong University. We used the guidelines of the Animal Care and Use Committee of Shandong University to conduct all animal experiments.

Expression and purification of MX1 VLP

The pET28-MX1-CP recombinant plasmid constructed based on Gene ID 1261502 was transformed into E. coli BL21 (DE3) competent cells. The cells were then coated on Kanamycin-resistant LB agar plates and incubated at 37 °C for 12 h. A single colony of BL21 (DE3) with the plasmid was picked into 10 mL of LB medium containing 50 μg/mL Kanamycin. The culture was grown at 37 °C for 12 h at a shaking speed of 220 rpm. Afterward, the resulting cloudy culture was transferred to the LB medium (1 L) with Kanamycin. The culture was continued at the same condition until the OD_{600} reached 0.6−0.8. Isopropyl *β*-D-1-thiogalactopyranoside (IPTG) was added to the culture to induce VLP expression (the final concentration of IPTG, 0.2 mM). The culture was grown at 16 °C for 16 h with a shaking speed of 220 rpm. The bacteria were then pelleted by centrifuging at 8000 rpm for 10 min. The pellets were resuspended in 0.1 M K-Phos buffer (pH 7.4). The bacteria in the suspension were then lysed with a

probe sonicator (a power of 50% for 10 min with intervals of 2 s pulses and 4 s stops, 2 to 3 times) in an ice bath. The lysis was centrifuged at 12000 rpm for 30 min. The supernatant containing MX1 VLP was incubated with 10% (w/v) PEG8000 overnight at 4 °C to allow protein precipitation. The mixture was centrifuged at 12,000 rpm for 10 min. The pellet was then resuspended in 0.1 M K-Phos buffer at pH = 7.4. The resuspended solution was mixed with chloroform/n-butanol (1:1, v/v) at a 1:1 (v/v) ratio until the mixture became colloidal. The colloidal mix was centrifuged at 12,000 rpm for 10 min to separate the layers. The top (aqueous) layer was collected. Viral capsid protein in the collected aqueous layer was concentrated down through Millipore 100 KDa MWCO ultrafiltration tubes and further purified by sucrose density gradients 10−40% (w/v). The crude MX1 VLP-loaded sucrose gradient was centrifuged with an Optima XPN-100 (Beckman) ultracentrifuge at 28,000 rpm for 4 h. The LED light shines through the top of the tube. The bright blue band from scattering light was collected as fractions of 1 mL. The collected fractions (~15 mL) were analyzed for purity of the capsid by size-exclusion HPLC (SEC) with a TSKgel SuperSW3000 column. The remaining sucrose in the collected fraction was removed by 100 kDa Amicon ultrafiltration. The total protein concentration in the final solution was determined by the Bradford method using bovine serum albumin as a standard. Purified MX1 VLPs were characterized by SEC (**Fig. S1**), SDS-PAGE (**Fig. S2**), MALDI-TOF-MS (**Fig. S3**), TEM (**Fig. 1** in the main text), and DLS (**Fig. S4**).

Synthesis of NHAcGD2-isothiocyanate (NCS) 1

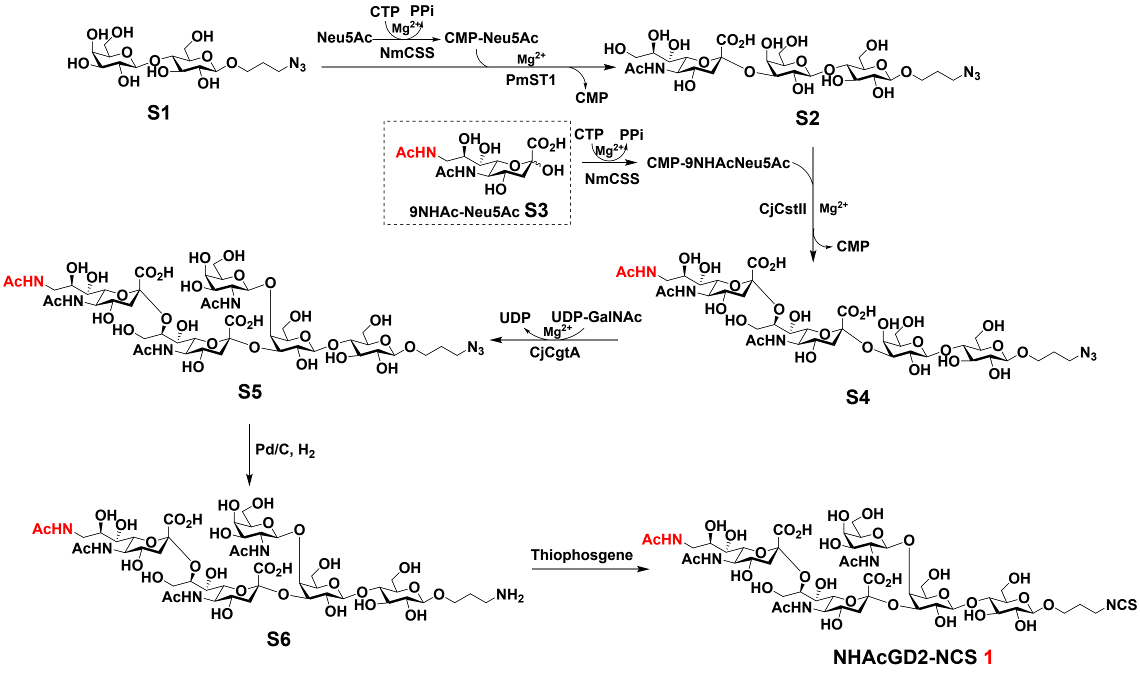

Scheme S1 Synthesis of NHAcGD2-NCS **1**.

3-Azidopropyl β-D-galactopyranosyl-(1→4)-β-D-glucopyranoside (LacβProN$_3$, S1)

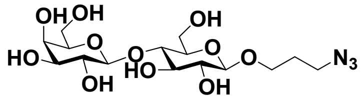

S1

The chemical synthesis of LacβProN$_3$ **S1** was detailed elsewhere.[5] ^{1}H NMR (600 MHz, D_2O) δ 4.44 (d, J = 8.0 Hz, 1H), 4.40 (d, J = 7.8 Hz, 1H), 3.99 – 3.92 (m, 2H), 3.88 (d, J = 3.4 Hz, 1H), 3.78 – 3.65 (m, 5H), 3.64 – 3.59 (m, 3H), 3.57 – 3.53 (m, 1H), 3.50 (dd, J = 10.0, 7.8 Hz, 1H), 3.42 (t, J = 6.7 Hz, 2H), 3.31 – 3.23 (m, 1H), 1.87 (p, J = 6.6 Hz, 2H); ^{13}C NMR (150 MHz, D_2O) δ 102.78, 101.96, 78.16, 75.21, 74.62, 74.21, 72.65, 72.35, 70.79, 68.41, 67.21, 60.88, 59.88, 47.69, 28.08. HRMS (ESI) m/z calcd for $C_{15}H_{27}N_3O_{11}Na$ [M + Na$^+$] 448.1538, found 448.1607.

3-Azidopropyl 5-acetamido-3,5-dideoxy-D-glycero-α-D-*galacto*-2-nonulopyranosyl-(2→3)-β-D-galactopyranosyl-(1→4)-β-D-glucopyranoside (GM3-N$_3$, S2)

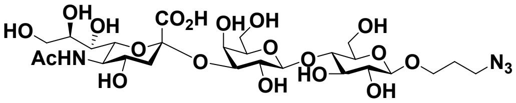

S2

To Tris-HCl buffer (100 mM, pH 8.5, 20 mL) containing $MgCl_2$ (20 mM) was added **S1** (170 mg, 0.4 mmol), Neu5Ac (186 mg, 0.6 mmol), and cytidine-5'-triphosphate (CTP, 316 mg, 0.6 mmol). Then, NmCSS (15 mg) and PmST1 (12 mg) were added to the reaction solution. The reaction was performed in an incubator at 37 °C, shaking at 100 rpm, and monitored by TLC (EtOAc/MeOH/H_2O/HOAc = 9:3:2:1, v/v). After the reaction was complete (~12 h), an equal volume of cold ethanol was added to the reaction mixture and incubated at 4 °C for 30 min. Afterward, the enzyme precipitate was removed by centrifugation. The supernatant was concentrated and subjected to BioGel P-2 gel column chromatography (eluted with H_2O), DEAE Sepharose fast flow (eluted with 0.05 M NaCl solution), and second BioGel P-2 gel column chromatography (eluted with H_2O) to give GM3-N_3 **S2** (265 mg, 92%). ^1H NMR (600 MHz, D_2O) δ 4.49 (d, J = 7.9 Hz, 1H), 4.44 (d, J = 8.0 Hz, 1H), 4.07 (dd, J = 9.9, 3.2 Hz, 1H), 3.98 – 3.89 (m, 3H), 3.87 – 3.76 (m, 4H), 3.74 – 3.51 (m, 12H), 3.42 (t, J = 6.7 Hz, 2H), 3.27 (t, J = 8.5 Hz, 1H), 2.71 (dd, J = 12.5, 4.6 Hz, 1H), 1.99 (s, 3H), 1.87 (p, J = 6.6 Hz, 2H), 1.76 (t, J = 12.1 Hz, 1H); ^{13}C NMR (150 MHz, D_2O) δ 174.84, 173.78, 102.47, 101.98, 99.63, 78.02, 75.33, 75.02, 74.63, 74.19, 72.71, 72.65, 71.62, 69.22, 68.23, 67.91, 67.32, 67.21, 62.40, 60.89, 59.87, 51.52, 47.70, 39.47, 28.08, 21.88. HRMS (ESI) m/z calcd for $C_{26}H_{43}N_4O_{19}$ $[M-H]^-$ 715.2527, found 715.2429.

3-Azidopropyl 5,9-acetamido-3,5-dideoxy-D-glycero-α-D-*galacto*-2-nonulopyranosyl-(2→8)-5-acetamido-3,5-dideoxy-D-glycero-α-D-*galacto*-2-nonulopyranosyl-(2→3)-β-D-galactopyranosyl-(1→4)-β-D-glucopyranoside (NHAcGD3-N_3, S4)

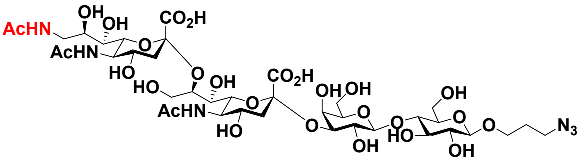

S4

To Tris-HCl buffer (100 mM, pH 8.5, 10 mL) containing $MgCl_2$ (20 mM) was added GM3-N_3 **S2** (143 mg, 0.2 mmol), 9-NHAc-Neu5Ac **S3** (84 mg, 0.24 mmol), and CTP (158 mg, 0.3 mmol). Then, NmCSS (3 mg), CjCstII (7.2 mg) were added to the reaction solution. The reaction was performed in an incubator at 37 ^oC, shaking at 100 rpm, and monitored by TLC (EtOAc/MeOH/H_2O/HOAc = 6:3:2:1, v/v). After the reaction was complete (~7 h), an equal volume of cold ethanol was added to the reaction mixture and incubated at 4 °C for 30 min. After centrifugation, the supernatant was concentrated and subjected to BioGel P-2 gel column chromatography (eluted with H_2O), DEAE Sepharose fast flow (eluted with 0.05 M NaCl solution), and second BioGel P-2 gel column chromatography (eluted with H_2O) to give NHAcGD3-N_3 **S4** (169 mg, 81%). ^{1}H NMR (600 MHz, D_2O) δ 4.51 (d, J = 7.9 Hz, 1H), 4.47 (d, J = 8.0 Hz, 1H), 4.18 – 4.11 (m, 2H), 4.08 (ddd, J = 9.9, 3.2, 1.6 Hz, 1H), 4.03 – 3.88 (m, 4H), 3.84 – 3.43 (m, 21H), 3.29 (ddd, J = 12.2, 8.1, 2.4 Hz, 2H), 2.75 (dd, J = 12.4, 4.6 Hz, 1H), 2.65 (dd, J = 12.3, 4.4 Hz, 1H), 2.04 (s, 3H), 2.02 (s, 3H), 2.01 (s, 3H), 1.89 (p, J = 6.6 Hz, 2H), 1.73 (td, J = 12.1, 7.3 Hz, 2H); ^{13}C NMR (150 MHz, D_2O) δ 174.87, 174.45, 173.36, 173.28, 102.58, 102.05, 100.32, 100.27, 78.13, 77.93, 75.34, 75.09, 74.71, 74.19, 74.08, 72.74, 72.36, 69.72, 69.59, 69.24, 69.21, 68.41, 67.89, 67.47, 67.26, 61.50, 60.98, 59.89, 59.21, 52.16, 51.60, 47.77, 42.21, 40.36, 39.45, 28.14, 22.19, 21.93, 21.72. HRMS (ESI) m/z calcd for $C_{39}H_{63}N_6O_{27}$ [M-H]$^-$ 1047.3747, found 1047.3612.

3-Azidopropyl 2-acetamido-2-deoxy-β-D-galactopyranosyl-(1→4)-[5,9-acetamido-3,5-dideoxy-D-glycero-α-D-*galacto*-2-nonulopyranosyl-(2→8)-5-acetamido-3,5-dideoxy-D-glycero-α-D-*galacto*-2-nonulopyranosyl-(2→3)]-β-D-galactopyranosyl-(1→4)-β-D-glucopyranoside (NHAcGD2-N_3, S5)

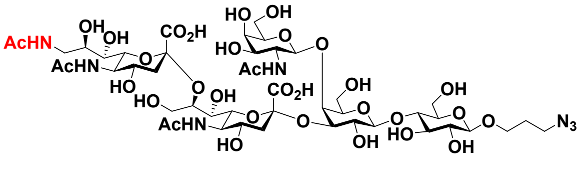

S5

To Tris-HCl buffer (100 mM, pH 7.5, 3 mL) containing $MgCl_2$ (20 mM) was added **S4** (24 mg, 0.023 mmol), UDP-GalNAc (23 mg, 0.036 mmol). Then, CjCgtA

(4.5 mg) was added to the reaction solution. The reaction was carried out in an incubator at 30 °C, shaking at 100 rpm, and monitored by TLC (EtOAc/MeOH/H_2O/HOAc = 6:3:2:1, v/v). After the reaction was complete (~2 h), an equal volume of cold ethanol was added to the reaction mixture and incubated at 4 °C for 30 min. After centrifugation, the supernatant was concentrated and subjected to BioGel P-2 gel column chromatography (eluted with H_2O), DEAE Sepharose fast flow (eluted with 0.05 M NaCl solution), and second BioGel P-2 gel column chromatography (eluted with H_2O) to give NHAcGD2-N_3 **S5** (23.9 mg, 83%). ^{1}H NMR (600 MHz, D_2O) δ 4.67 (d, J = 8.4 Hz, 1H), 4.47 (dd, J = 10.1, 7.9 Hz, 2H), 4.19 – 4.05 (m, 3H), 4.01 (d, J = 2.8 Hz, 1H), 3.99 – 3.92 (m, 2H), 3.91 – 3.41 (m, 27H), 3.37 (dd, J = 10.0, 7.9 Hz, 1H), 3.34 – 3.23 (m, 2H), 2.73 (dd, J = 12.4, 4.6 Hz, 1H), 2.65 (dd, J = 12.4, 4.4 Hz, 1H), 2.03 (s, 3H), 2.01 (s, 3H), 2.00 (s, 3H), 1.99 (s, 3H), 1.88 (p, J = 6.7 Hz, 2H), 1.80 – 1.67 (m, 2H); ^{13}C NMR (150 MHz, D_2O) δ 174.81, 174.76, 174.40, 173.26, 173.19, 102.64, 102.02, 100.45, 78.26, 78.15, 75.83, 74.69, 74.41, 74.34, 74.16, 74.14, 73.63, 72.66, 72.37, 70.75, 69.72, 69.60, 69.54, 69.12, 68.38, 68.04, 67.60, 67.23, 61.32, 60.81, 60.49, 59.88, 59.19, 57.31, 52.38, 52.25, 51.60, 47.76, 42.16, 40.30, 39.04, 28.12, 22.43, 22.20, 21.91, 21.73, 16.66. HRMS (ESI) m/z calcd for $C_{47}H_{76}N_7O_{32}$ [M-H]$^-$ 1250.4540, found 1250.4379.

NHAcGD2-NH_2 S6

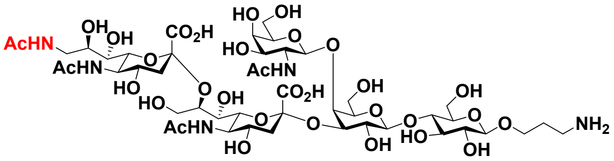

S6

S5 (24 mg, 0.019 mmol) was dissolved in H_2O/MeOH (2 mL, 1:1 v/v). To this solution was added Palladium on carbon (Pd/C, 10 wt.% loading, 12 mg). The atmosphere was removed by vacuum and replaced by H_2, and the reaction solution was stirred under H_2 for 12 h. Then, the reaction solution was filtered, concentrated, and purified by BioGel P-2 gel column chromatography (eluted with H_2O) to obtain pure **S6** (20 mg, 86%). ^{1}H NMR (600 MHz, D_2O) δ 4.67 (d, J = 8.4 Hz, 1H), 4.48 (t, J = 7.5

Hz, 2H), 4.19 – 3.97 (m, 6H), 3.96 – 3.45 (m, 27H), 3.42 – 3.36 (m, 1H), 3.30 (dd, *J* = 14.2, 8.1 Hz, 2H), 3.15 (h, *J* = 6.4 Hz, 2H), 2.74 (dd, *J* = 12.4, 4.6 Hz, 1H), 2.68 (dd, *J* = 12.4, 4.4 Hz, 1H), 2.04 (s, 3H), 2.03 (s, 3H), 2.01 (s, 3H), 2.01 (s, 3H), 1.79 – 1.67 (m, 2H); ^{13}C NMR (150 MHz, D_2O) δ 174.84, 174.76, 174.41, 173.23, 173.14, 102.67, 102.02, 100.48, 100.31, 78.26, 78.07, 75.63, 74.72, 74.50, 74.36, 74.22, 74.11, 73.64, 72.63, 72.40, 70.70, 69.74, 69.53, 69.11, 68.37, 67.98, 67.79, 67.59, 61.34, 60.83, 60.52, 59.80, 52.38, 52.26, 51.61, 42.15, 40.32, 39.22, 37.80, 37.52, 26.56, 26.41, 22.45, 22.21, 21.93, 21.74. HRMS (ESI) m/z calcd for $C_{47}H_{81}N_5O_{32}$ $[M+2H]^{2+}$ 613.7427, found 613.7563.

NHAcGD2-NCS 1

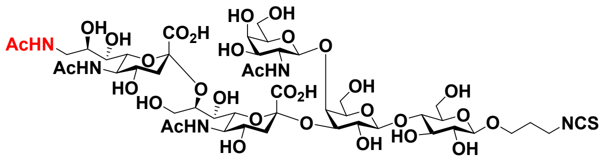

1

S6 (18 mg, 0.015 mmol) was dissolved in an aqueous $NaHCO_3$ solution (500 µL, 10 mg/mL). To the solution was added chloroform (750 µL) containing thiophosgene (1.67 µL, 21.8 µmol). The reaction solution was stirred at room temperature (RT). After ~3 h, the spot of **S6** disappeared entirely. The reaction mixture was then diluted with water. The aqueous layer was extracted twice with chloroform and freeze-dried to give NHAcGD2-NCS **1** without further purification. ^{1}H NMR (600 MHz, D_2O) δ 4.62 (d, *J* = 8.4 Hz, 1H), 4.43 (dd, *J* = 7.8, 5.3 Hz, 2H), 4.14 – 3.99 (m, 3H), 3.99 – 3.85 (m, 4H), 3.85 – 3.39 (m, 28H), 3.33 (dd, *J* = 9.9, 7.9 Hz, 1H), 3.25 (dd, *J* = 14.2, 8.2 Hz, 2H), 2.69 (dd, *J* = 12.4, 4.6 Hz, 1H), 2.62 (dd, *J* = 12.4, 4.5 Hz, 1H), 1.99 (s, 3H), 1.97 (s, 3H), 1.96 (s, 3H), 1.95 (s, 3H), 1.74 – 1.63 (m, 2H). HRMS (ESI) m/z calcd for $C_{48}H_{75}N_5O_{32}S$ $[M-2H]^{2-}$ 632.7063, found 632.6980; $C_{48}H_{76}N_5O_{32}S$ $[M-H]^{-}$ 1266.4200, found 1266.4038.

Synthesis of GD2-NCS 5

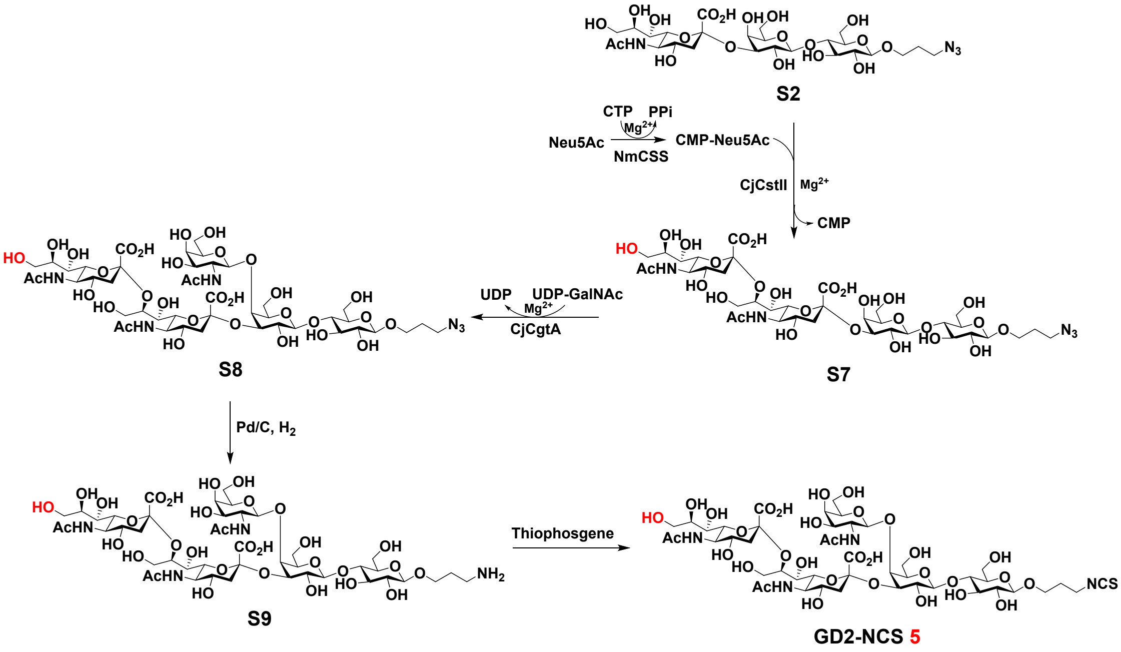

Scheme S2 Synthesis of GD2-NCS **5**.

GD3-N_3 S7

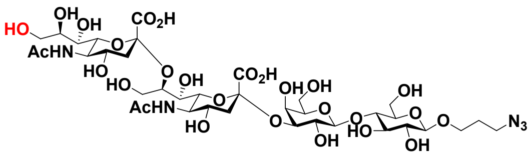

To Tris-HCl buffer (100 mM, pH 8.5, 10 mL) containing $MgCl_2$ (20 mM) was added **S2** (143 mg, 0.2 mmol), Neu5Ac (74 mg, 0.24 mmol), and CTP (158 mg, 0.3 mmol). Then, NmCSS (1.5 mg) and CjCstII (3.6 mg) were added to the reaction solution. The reaction was performed in an incubator at 30 ^{o}C with shaking at a speed of 100 rpm. TLC was used to monitor the reaction (EtOAc/MeOH/H_2O/HOAc = 9:3:2:1, v/v). When the reaction was complete (~1 h), an equal volume of cold ethanol was added to the mixture. After incubating at 4 °C for 30 min, the reaction solution was centrifuged. The resulting supernatant was concentrated and subjected to BioGel P-2 gel column chromatography (eluted with H_2O), DEAE Sepharose fast flow (eluted with 0.05 M NaCl solution), and second BioGel P-2 gel column chromatography (eluted with H_2O) to give **S7** (125 mg, 62%). ^{1}H NMR (600 MHz, D_2O) δ 4.50 (d, J = 8.4 Hz,

1H), 4.46 (d, J = 8.0 Hz, 1H), 4.19 – 4.04 (m, 3H), 4.00 – 3.54 (m, 24H), 3.44 (t, J = 6.9 Hz, 2H), 3.29 (t, J = 8.6 Hz, 1H), 2.75 (dd, J = 12.4, 4.6 Hz, 1H), 2.65 (dd, J = 12.3, 4.4 Hz, 1H), 2.04 (s, 3H), 2.01 (s, 3H), 1.89 (p, J = 6.6 Hz, 2H), 1.72 (t, J = 12.1 Hz, 2H); ^{13}C NMR (150 MHz, D_2O) δ 174.89, 173.27, 102.58, 102.05, 100.40, 100.09, 78.14, 77.91, 75.37, 75.13, 74.72, 74.19, 73.92, 72.75, 72.56, 71.65, 69.20, 68.41, 68.03, 67.84, 67.36, 67.26, 62.46, 61.47, 61.01, 59.89, 52.18, 51.64, 47.78, 40.41, 39.60, 28.14, 22.22, 21.94. HRMS (ESI) m/z calcd for $C_{37}H_{60}N_5O_{27}$ [M-H]$^-$ 1006.3481, found 1006.3065.

GD2-N$_3$ S8

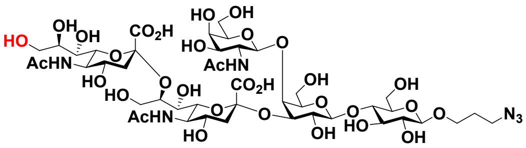

S8

To Tris-HCl buffer (100 mM, pH 7.5, 3 mL) containing $MgCl_2$ (20 mM) was added **S7** (24 mg, 0.024 mmol), UDP-GalNAc (23 mg, 0.038 mmol). Then, CjCgtA (4.5 mg) was added to the reaction solution. The reaction was performed in an incubator at 30 °C with shaking at a speed of 100 rpm for 2 h. Afterward, an equal volume of cold ethanol was added to the mixture. After incubating at 4 °C for 30 min, the reaction solution was centrifuged. The resulting supernatant was concentrated and subjected to BioGel P-2 gel column chromatography (eluted with H_2O), DEAE Sepharose fast flow (eluted with 0.05 M NaCl solution), and second BioGel P-2 gel column chromatography (eluted with H_2O) to give **S8** (26 mg, 89%). ^{1}H NMR (600 MHz, D_2O) δ 4.69 (d, J = 8.4 Hz, 1H), 4.48 (dd, J = 10.2, 7.9 Hz, 2H), 4.21 – 4.07 (m, 3H), 4.03 – 3.96 (m, 2H), 3.92 – 3.52 (m, 27H), 3.45 (td, J = 6.6, 4.3 Hz, 2H), 3.39 (ddd, J = 10.1, 7.9, 2.8 Hz, 1H), 3.32 – 3.25 (m, 1H), 2.75 (dd, J = 12.4, 4.7 Hz, 1H), 2.66 (dd, J = 12.4, 4.5 Hz, 1H), 2.06 (s, 3H), 2.03 (s, 3H), 2.01 (s, 3H), 1.90 (p, J = 6.5 Hz, 2H), 1.81 – 1.68 (m, 2H); ^{13}C NMR (150 MHz, D_2O) δ 174.87, 174.84, 174.77, 173.30, 173.26, 102.63, 102.02, 100.39, 78.24, 75.87, 74.70, 74.40, 74.34, 74.15, 73.62, 72.66, 72.54, 71.64, 70.76, 69.61, 69.16, 68.41, 68.00, 67.61, 67.24, 62.43, 61.37, 60.82, 60.52, 59.89, 52.37, 52.26, 51.64, 48.01, 47.77, 40.38, 39.01, 28.13, 22.43, 22.23, 21.92.

HRMS (ESI) m/z calcd for $C_{45}H_{72}N_6O_{32}$ $[M-2H]^{2-}$ 604.2101, found 604.1873.

GD2-NH$_2$ S9

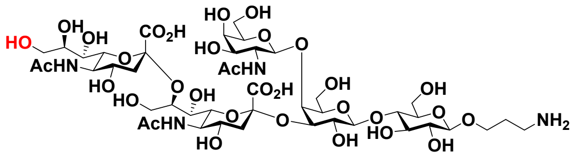

S9

S8 (24 mg, 0.02 mmol) was dissolved in H_2O/MeOH (2 mL, 1:1 v/v). To this solution was added Pd/C (10 wt.% loading, 12 mg). The atmosphere was removed by vacuum and replaced by H_2, and the reaction solution was stirred under H_2 for 12 h. Then, the reaction solution was filtered, concentrated, and purified by BioGel P-2 gel column chromatography (eluted with H_2O) to obtain pure **S9** (18 mg, 76%). ^{1}H NMR (600 MHz, D_2O) δ 4.61 (d, *J* = 8.4 Hz, 1H), 4.43 (t, *J* = 7.1 Hz, 2H), 4.15 – 3.90 (m, 6H), 3.85 – 3.50 (m, 28H), 3.33 (t, *J* = 8.9 Hz, 1H), 3.24 (t, *J* = 8.4 Hz, 1H), 3.08 (t, *J* = 6.9 Hz, 2H), 2.69 (dd, *J* = 12.2, 4.5 Hz, 1H), 2.64 – 2.59 (m, 1H), 1.99 (s, 3H), 1.97 (s, 3H), 1.95 (s, 3H), 1.73 – 1.62 (m, 2H). HRMS (ESI) m/z calcd for $C_{45}H_{74}N_4O_{32}$ $[M-2H]^{2-}$ 591.2149, found 591.2100; $C_{45}H_{75}N_4O_{32}$ $[M-H]^-$ 1183.4370, found 1183.4228.

GD2-NCS 5

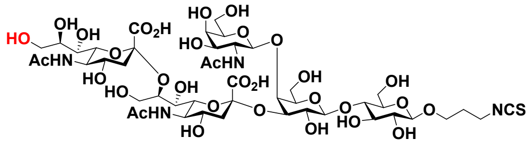

5

S9 (18 mg, 0.015 mmol) was dissolved in an aqueous $NaHCO_3$ solution (500 μL, 10 mg/mL). To the solution was added chloroform (750 μL) containing thiophosgene (1.67 μL, 21.8 μmol). The reaction solution was stirred at RT for 3 h. The reaction mixture was then diluted with water. The aqueous layer was extracted twice with chloroform and freeze-dried to give GD2-NCS **5**. ^{1}H NMR (600 MHz, D_2O) δ 4.64 (d, *J* = 8.4 Hz, 1H), 4.45 (dd, *J* = 8.1, 4.7 Hz, 2H), 4.18 – 3.92 (m, 6H), 3.90 – 3.46 (m, 30H), 3.35 (t, *J* = 8.8 Hz, 1H), 3.26 (t, *J* = 8.2 Hz, 1H), 2.71 (dd, *J* = 12.4, 4.7 Hz, 1H), 2.63 (dd, *J* = 12.7, 4.3 Hz, 1H), 2.02 (s, 3H), 1.99 (s, 3H), 1.98 (s, 3H), 1.76 – 1.64 (m, 2H). HRMS (ESI) m/z calcd for $C_{46}H_{72}N_4O_{32}S$ $[M-2H]^{2-}$ 612.1931, found 612.1900.

Synthesis of NHAcGD3-NCS 8 and GD3-NCS 11

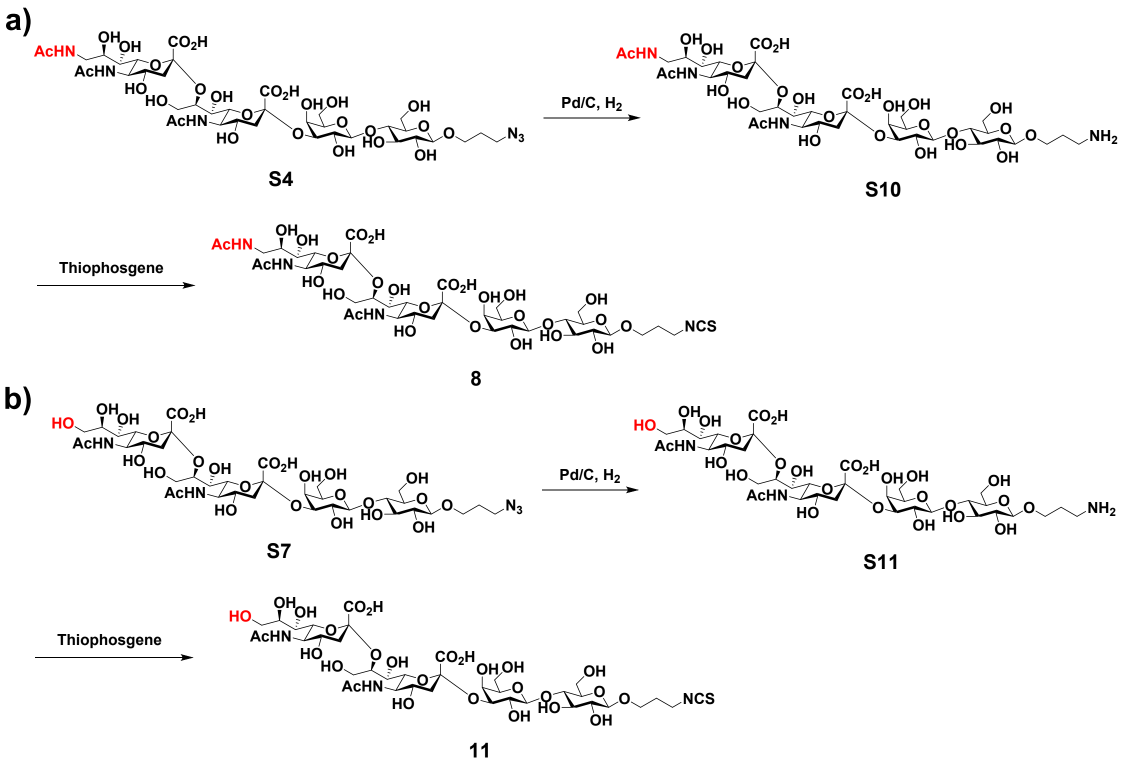

Scheme S3 Synthesis of (a) NHAcGD3-NCS **8** and (b) GD3-NCS **11**.

NHAcGD3-NH2 S10

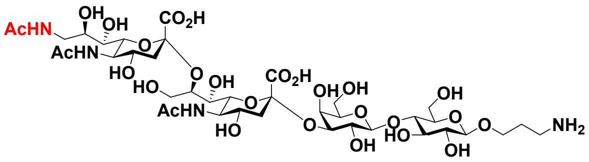

S10

S4 (20 mg, 0.02 mmol) was dissolved in H_2O/MeOH (2 mL, 1:1 v/v). To this solution was added Pd/C (10 wt.% loading, 10 mg). The atmosphere was removed by vacuum and replaced by H_2, and the reaction solution was stirred under H_2 for 12 h. Then, the reaction solution was filtered, concentrated, and purified by BioGel P-2 gel column chromatography (eluted with H_2O) to obtain pure NHAcGD3-NH_2 **S10** (15 mg, 71%). ^1H NMR (600 MHz, D_2O) δ 4.50 (dd, J = 8.0, 5.5 Hz, 2H), 4.19 – 3.87 (m, 8H), 3.86 – 3.50 (m, 19H), 3.46 (dd, J = 9.2, 1.9 Hz, 1H), 3.36 – 3.25 (m, 2H), 3.15 (t, J =

7.2 Hz, 2H), 2.76 (dd, J = 12.4, 4.6 Hz, 1H), 2.66 (dd, J = 12.3, 4.4 Hz, 1H), 2.04 (s, 3H), 2.02 (s, 3H), 2.01 (s, 3H), 1.73 (td, J = 12.2, 6.4 Hz, 2H); ^{13}C NMR (150 MHz, D_2O) δ 174.85, 174.46, 173.27, 173.23, 102.64, 102.04, 100.51, 98.00, 78.08, 77.91, 75.40, 75.17, 74.72, 74.14, 74.00, 72.69, 72.40, 69.75, 69.56, 69.18, 69.09, 68.37, 67.82, 67.79, 67.45, 65.85, 61.42, 61.01, 59.81, 52.16, 51.61, 42.19, 40.38, 39.49, 37.51, 26.58, 22.19, 21.93, 21.73. HRMS (ESI) m/z calcd for $C_{39}H_{65}N_4O_{27}$ [M-H]$^-$ 1021.3842, found 1021.3723.

NHAcGD3-NCS 8

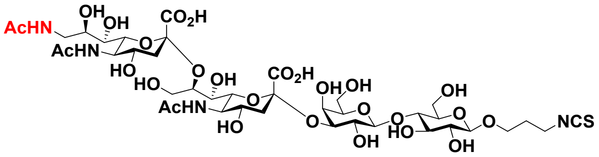

8

S10 (15 mg, 0.015 mmol) was dissolved in an aqueous $NaHCO_3$ solution (500 µL, 10 mg/mL). To the solution was added chloroform (750 µL) containing thiophosgene (1.67 µL, 21.8 µmol). The reaction solution was stirred at RT for 3 h. The reaction mixture was then diluted with water. The aqueous layer was extracted twice with chloroform and freeze-dried to give NHAcGD3-NCS **8**. ^{1}H NMR (600 MHz, D_2O) δ 4.31 – 4.20 (m, 2H), 3.96 – 3.82 (m, 3H), 3.81 – 3.21 (m, 27H), 3.16 – 2.99 (m, 2H), 2.51 (dd, J = 12.5, 4.5 Hz, 1H), 2.41 (dd, J = 12.3, 4.3 Hz, 1H), 1.80 (d, J = 1.6 Hz, 3H), 1.78 (s, 3H), 1.77 (s, 3H), 1.49 (q, J = 11.7 Hz, 2H). HRMS (ESI) m/z calcd for $C_{40}H_{63}N_4O_{27}S$ [M-H]$^-$ 1063.3406, found 1063.3273.

GD3-NH$_2$ S11

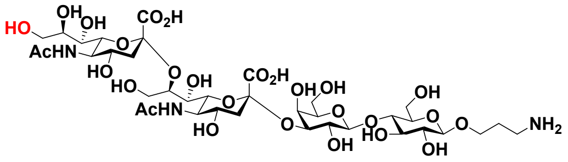

S11

S7 (20 mg, 0.02 mmol) was dissolved in H_2O/MeOH (2 mL, 1:1 v/v). To this solution was added Pd/C (10 wt.% loading, 10 mg). The atmosphere was removed by vacuum and replaced by H_2, and the reaction solution was stirred under H_2 for 12 h. Then, the reaction solution was filtered, concentrated, and purified by BioGel P-2 gel

column chromatography (eluted with H_2O) to obtain pure GD3-NH_2 **S11** (15 mg, 77%). ^{1}H NMR (600 MHz, D_2O) δ 4.50 (dd, *J* = 8.0, 6.2 Hz, 2H), 4.21 – 3.92 (m, 6H), 3.93 – 3.77 (m, 8H), 3.77 – 3.50 (m, 15H), 3.32 (t, *J* = 8.5 Hz, 1H), 3.14 (t, *J* = 7.0 Hz, 2H), 2.77 (dd, *J* = 12.4, 4.7 Hz, 1H), 2.66 (dd, *J* = 12.3, 4.4 Hz, 1H), 2.05 (s, 3H), 2.01 (s, 3H), 1.72 (t, *J* = 12.1 Hz, 2H). HRMS (ESI) m/z calcd for $C_{37}H_{62}N_3O_{27}$ $[M-H]^-$ 980.3576, found 980.3518.

GD3-NCS 11

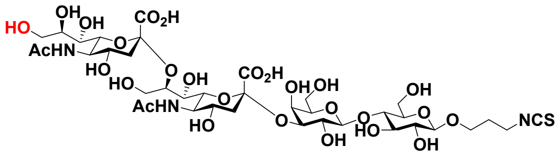

11

S11 (14.7 mg, 0.015 mmol) was dissolved in an aqueous $NaHCO_3$ solution (500 µL, 10 mg/mL). To the solution was added chloroform (750 µL) containing thiophosgene (1.67 µL, 21.8 µmol). The reaction solution was stirred at RT for 3 h. The reaction mixture was then diluted with water. The aqueous layer was extracted twice with chloroform and freeze-dried to give GD3-NCS **11**. ^{1}H NMR (600 MHz, D_2O) δ 4.52 – 4.39 (m, 2H), 4.19 – 4.00 (m, 3H), 4.00 – 3.48 (m, 28H), 3.27 (t, *J* = 8.8 Hz, 1H), 2.72 (d, *J* = 14.3 Hz, 1H), 2.62 (d, *J* = 11.9 Hz, 1H), 2.01 (s, 3H), 1.97 (s, 3H), 1.68 (t, *J* = 12.2 Hz, 2H). HRMS (ESI) m/z calcd for $C_{38}H_{60}N_3O_{27}S$ $[M-H]^-$ 1022.3140, found 1022.3038.

Synthesis of MX1-NHAcGD2 conjugate 2 and MX1-GD2 conjugate 4

To synthesize MX1-NHAcGD2 conjugate **2**, a solution of MX1 VLP (2 mg) in 0.1 M K-Phos buffer (pH 8.0, 0.2 mL) was cooled on ice, followed by the addition of NHAcGD2-NCS **1** (8 mg, 6.3 µmol, from 100 mg/mL stock solution in H_2O). To synthesize MX1-GD2 conjugate **4**, a solution of MX1 VLP (2 mg) in 0.1 M K-Phos buffer (pH 8.0, 0.2 mL) was cooled on ice, followed by the addition of GD2-NCS **5** (6.4 mg, 5.2 µmol, from 100 mg/mL stock solution in H_2O). The mixture was allowed to warm to RT and reacted at 37 ^oC for 12 h. The free NHAcGD2 or GD2 was removed by centrifugal filtration against 0.1 M K-Phos buffer (pH 7.4). MALDI-TOF MS was

used to determine the extent of particle modification (see **Figs. S5** and **S7**). The total protein concentration was determined by the Bradford assay.

Synthesis of CRM197-NHAcGD2 conjugate 3

CRM197 (2 mg, from 10 mg/mL stock solution in 0.1 M K-phos buffer, pH 8.0) was incubated with NHAcGD2-NCS **1** (2 mg, 1.5 μmol, from 100 mg/mL stock solution in H_2O). The tube was placed at 37 °C overnight. The mixture was purified using a 30 kDa Amicon ultra centrifugal filter tube (Millipore) against K-phos buffer to remove free NHAcGD2. The Bradford assay was employed to determine the final amounts of CRM197-NHAcGD2 recovered. MALDI-TOF MS was used to determine the number of NHAcGD2 per CRM197 protein (see **Fig. S6**).

Synthesis of MX1-NHAcGD3 conjugate 9 and MX1-GD3 conjugate 10

For the synthesis of MX1-NHAcGD3 (**9**) and MX1-GD3 (**10**), a solution of MX1 VLP (2 mg) in 0.1 M K-Phos buffer (pH 8.0, 0.2 mL) was cooled on ice, followed by the addition of NHAcGD3-NCS **8** (9 mg, 8.4 μmol, from 100 mg/mL stock solution) or GD3-NCS **11** (8.6 mg, 8.4 μmol, from 100 mg/mL stock solution). The mixture was allowed to warm to RT and reacted at 37 °C for 12 h. The free NHAcGD3 or GD3 was removed by centrifugal filtration against 0.1 M K-Phos buffer (pH 7.4). MALDI-TOF MS was used to determine the extent of particle modification (see **Figs. S10** and **S11**).

Synthesis of BSA-NHAcGD2 conjugate 6, BSA-GD2 conjugate 7, BSA-NHAcGD3 conjugate 12 and BSA-GD3 conjugate 13

For the synthesis of BSA-NHAcGD2 conjugate **6** and BSA-GD2 conjugate **7**, a solution of BSA (2 mg) in 0.1 M K-Phos buffer pH 8.0 (0.2 mL) was cooled on ice, followed by the addition of NHAcGD2-NCS **1** (2 mg, 1.5 μmol) or GD2-NCS **5** (1.8 mg, 1.5 μmol). For the synthesis of BSA-NHAcGD3 conjugate **12** and BSA-GD3 conjugate **13**, a solution of BSA (2 mg) in 0.1 M K-Phos buffer pH 8.0 (0.2 mL) was cooled on ice, followed by the addition of NHAcGD3-NCS **8** (1.6 mg, 1.5 μmol) or GD3-NCS **11** (1.5 mg, 1.5 μmol). The reaction was incubated at 37 °C overnight. The

product was purified by an Amicon Ultra 30 kDa MW cut-off against 0.1 M K-Phos buffer. Total protein content was quantified by the Bradford assay. MALDI-TOF MS was used to determine the extent of BSA modification (see **Figs. S8**, **S9**, **S12** and **S13**).

Mouse immunization

C57BL/6 female mice aged 6−8 weeks were subcutaneously injected under the scruff on day 0 with 0.2 mL of various vaccines (MX1, **2**−**4**, **9** or **10**) with MPLA (20 μL, 1 mg/mL in DMSO) as an adjuvant to evaluate their effectiveness. On days 14 and 28, the booster was given subcutaneously with the same dose. All vaccines administered have 6.6 nmol of carbohydrate antigen. Sera samples were collected on days 0 (before immunization) and day 35.

Enzyme-linked immunosorbent assay (ELISA)

To detect the levels of specific antibodies produced in the serum of mice immunized with MX1-/CRM197-NHAcGD2, MX1-GD2, MX1-NHAcGD3 and MX1-GD3 conjugates, the BSA-NHAcGD2/-GD2/-NHAcGD3/or-GD3 conjugates (**6**, **7**, **12** or **13**) in $NaHCO_3/Na_2CO_3$ buffer (0.05 M, pH 9.6) were respectively coated to Nunc MaxiSorp® 96-well plates (1 μg per well) overnight at 4 °C. The coated plates were washed with 0.5% Tween-20 (PBST) and incubated with 1% BSA at RT for 1 h to block the non-specific sites. Mouse serum was diluted by concentration gradient and incubated at 37 °C for 2 h. After washing with PBST, HRP-conjugated goat anti-mouse antibody (IgG, IgG1, IgG2b, IgG2c or IgG3) diluted at 1:2000 was added to wells and then incubated at 37 °C for 1 h. After washing with PBST, the plate was added 3,3',5,5'-tetramethylbenzidine (TMB) for color development. After 15 min, color development was terminated by adding 0.5 M H_2SO_4. Absorbance was measured at 450 nm.

Fluorescence-activated cell sorting (FACS)

EL4, IMR-32 or SK-MEL 28 cells were cultured at 37 °C under 5% CO_2 in cell culture medium. Suspensions of 3.0×10^5 cells were added to each 1.5 mL of microcentrifuge tubes, then centrifuged at 1,600 rpm for 5 min to remove the supernatant. The cell pellets were washed with buffer (1% FBS in PBS with 0.1 % NaN_3) and incubated with mouse sera (5 μL) in buffer (95 μL) for 30 min on ice. The incubated cells were washed twice with buffer and incubated with FITC conjugated goat anti-mouse IgG antibody (2 μL each) for 30 min on ice. The cells were washed

twice, re-suspended in buffer, and analyzed by flow cytometry.

Complement-dependent cytotoxicity (CDC) assay

IMR-32 or SK-MEL-28 cells (1×10^4 cells/well) were cultured in 96 well plates with MEM medium overnight. The medium was removed carefully. A dilution of mouse sera (1/20) from different groups of immunized mice in 50 μL of MEM (with 1% P.S) were respectively added to the plate and incubated for 30 min at 37 ^{0}C. Then, rabbit sera complement at a dilution (1/15) in 50 μL of MEM medium (with 10% FBS and 1% P.S.) was added and incubated at 37 °C for 3 h. MTS was then added into each well and incubated further at 37 °C for 3 h. The absorption was measured at 490 nm. Cytotoxicity was calculated according to the method in the literature.[6]

Product Characterization Spectra

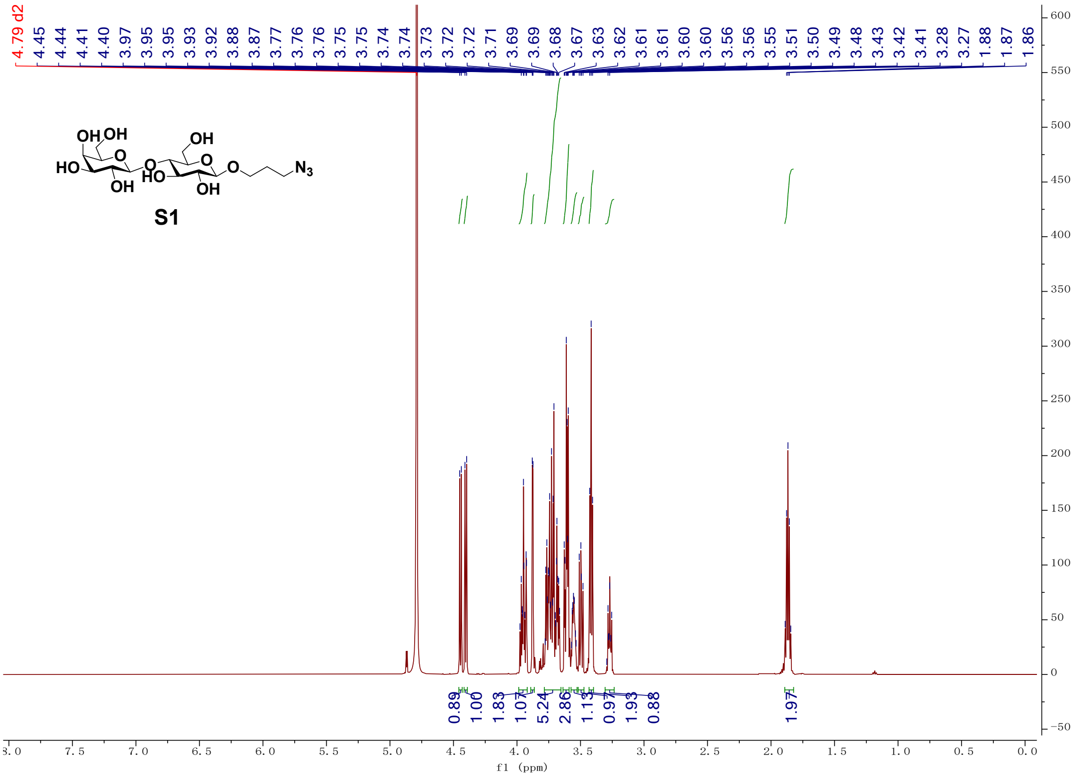

^{1}H-NMR of **S1** (600 MHz, D_2O)

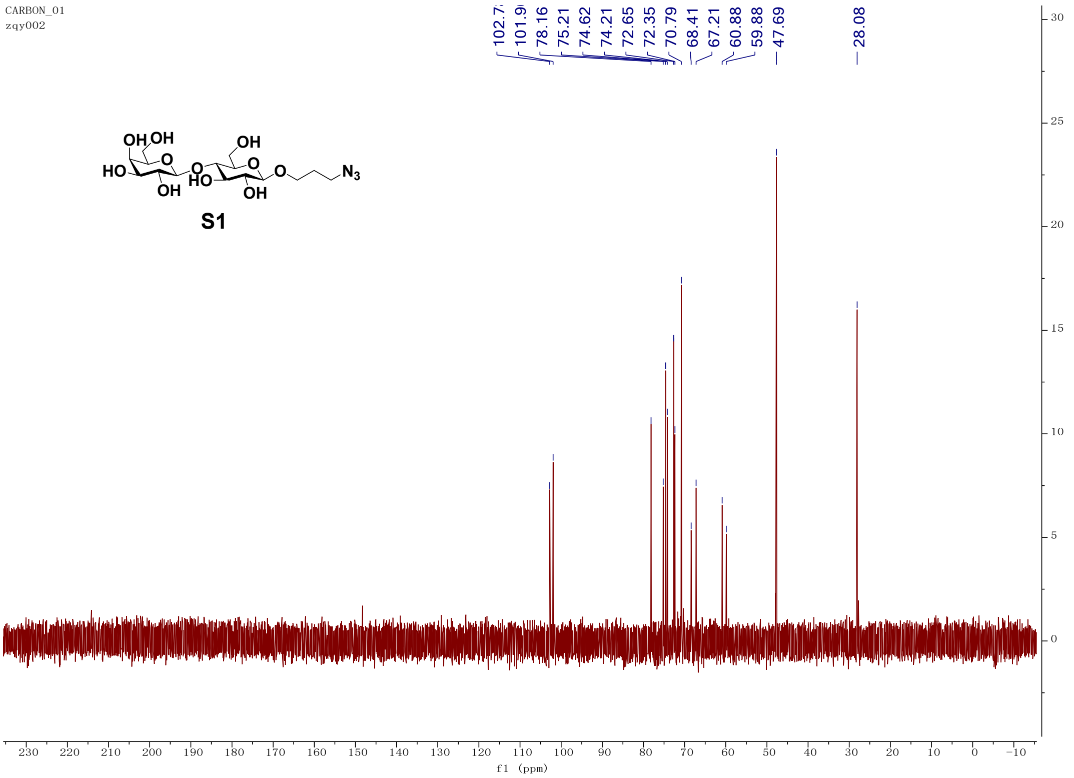

^{13}C-NMR of **S1** (150 MHz, D_2O)

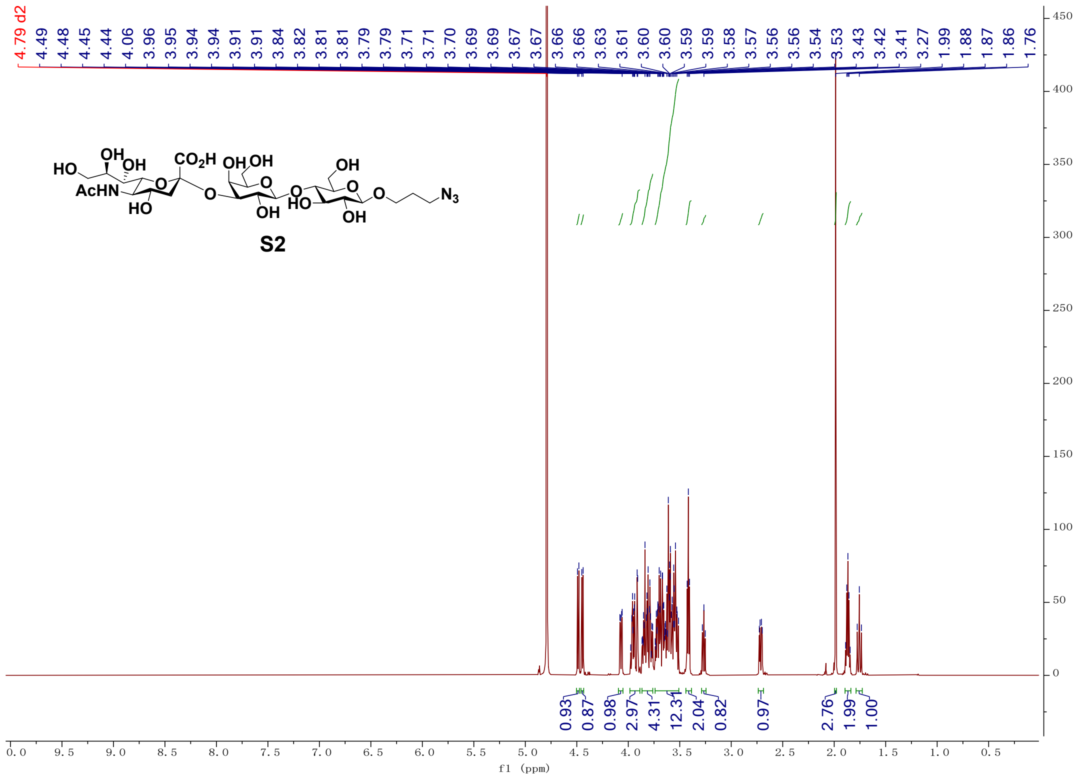

^{1}H-NMR of **S2** (600 MHz, D_2O)

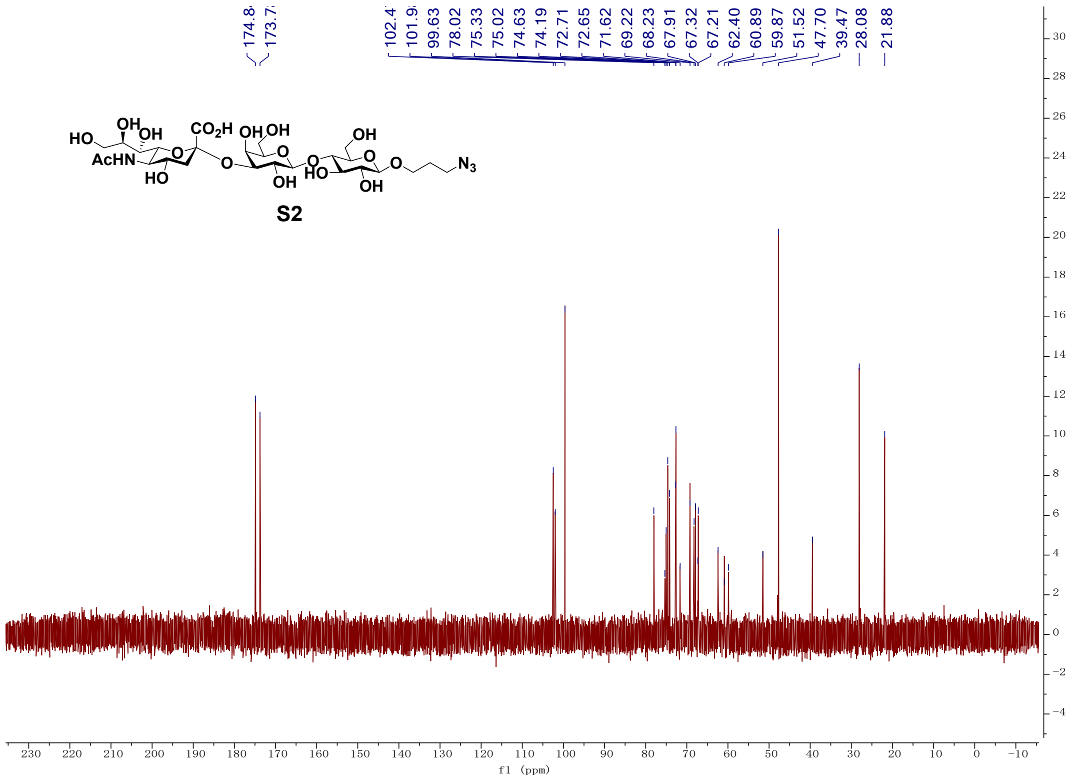

^{13}C-NMR of **S2** (150 MHz, D_2O)

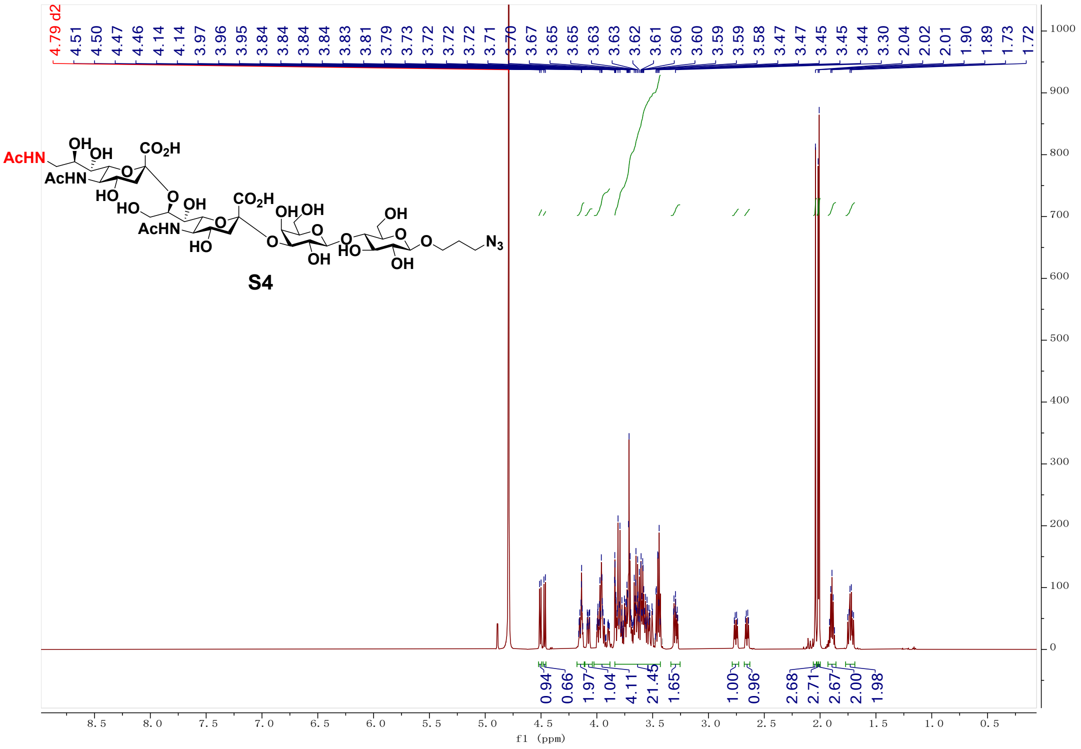

^{1}H-NMR of **S4** (600 MHz, D_2O)

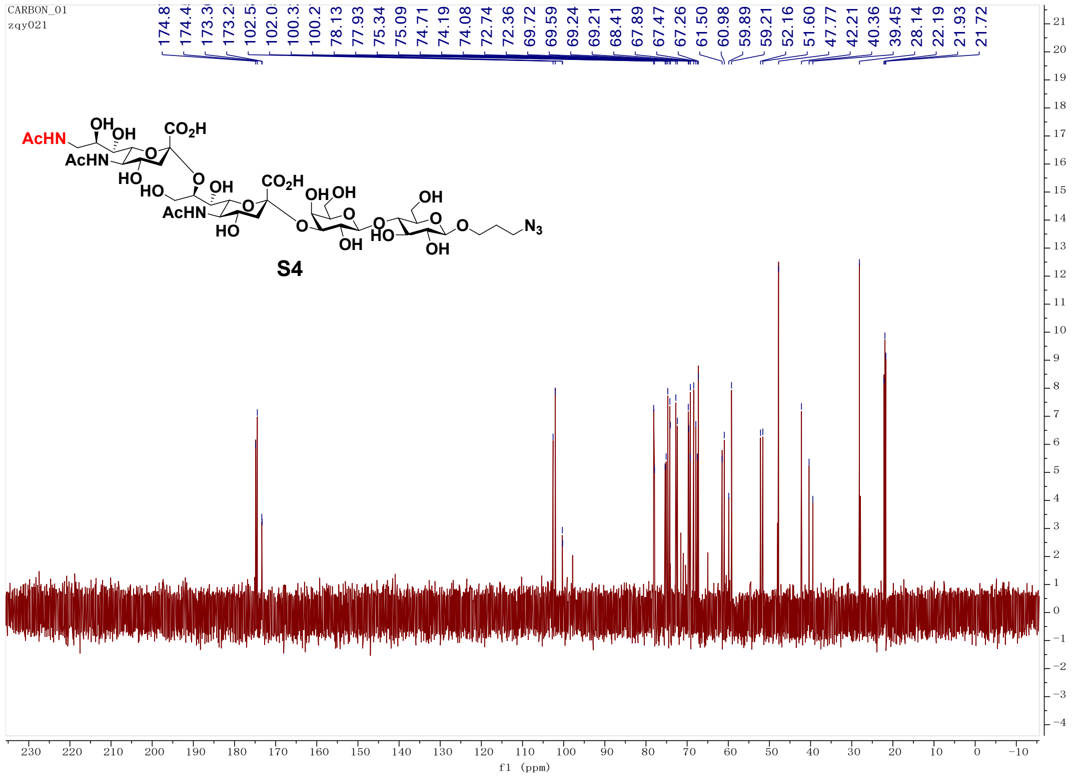

^{13}C-NMR of **S4** (150 MHz, D_2O)

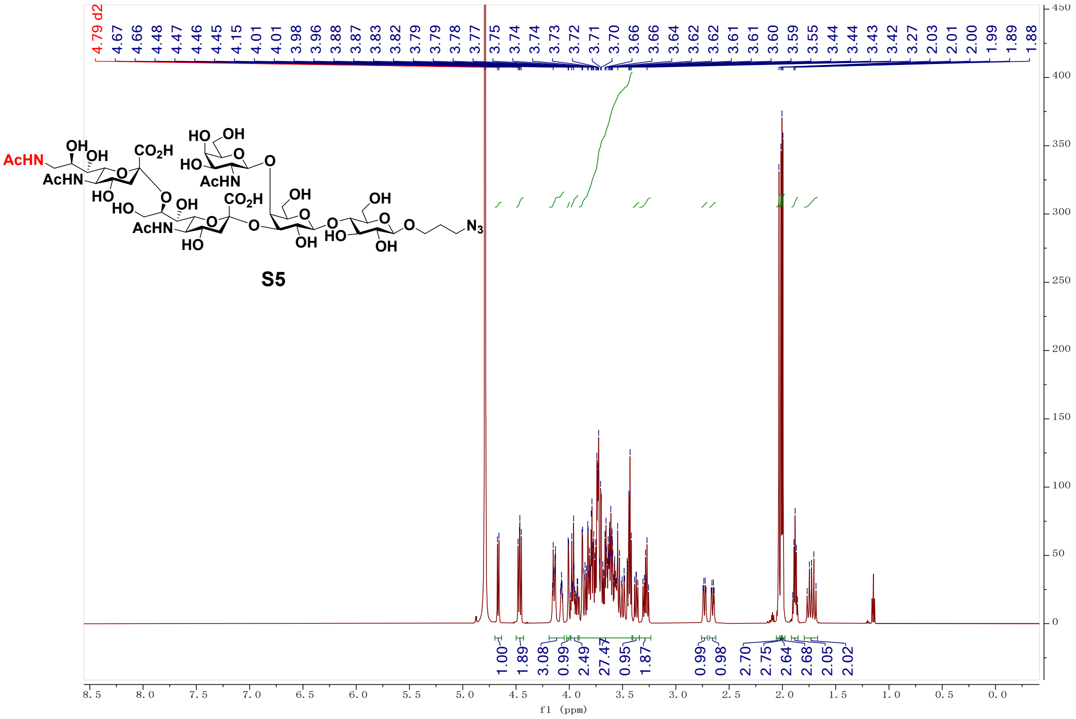

^{1}H-NMR of **S5** (600 MHz, D_2O)

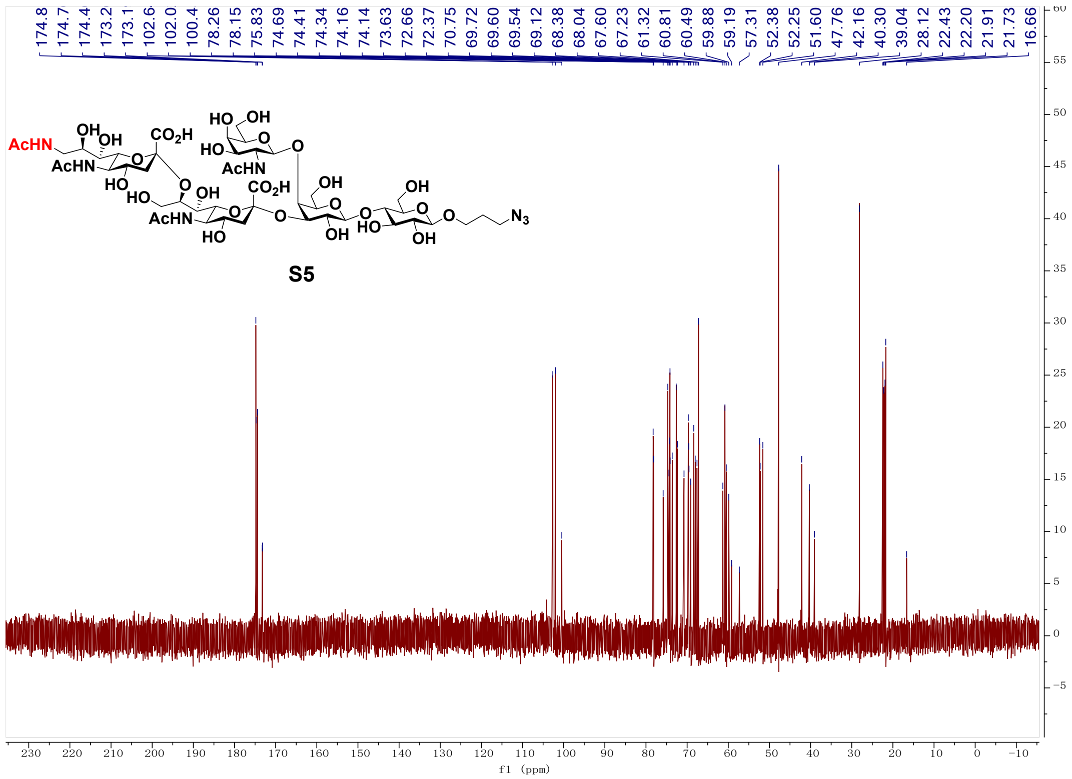

^{13}C-NMR of **S5** (150 MHz, D_2O)

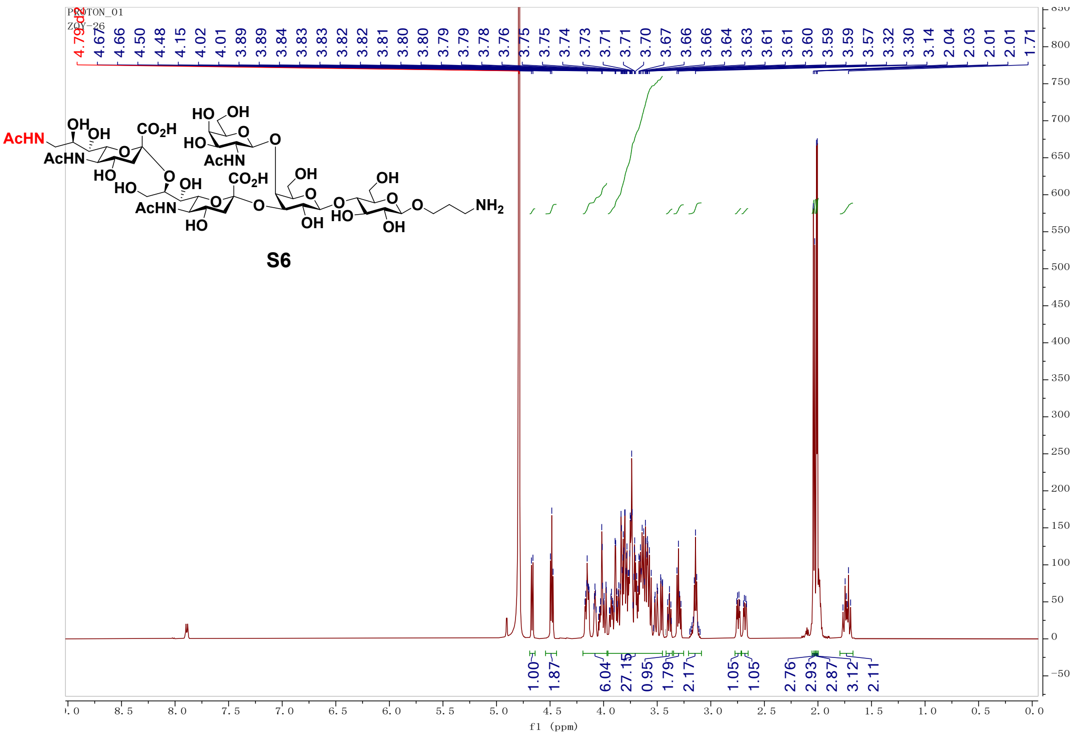

^{1}H-NMR of **S6** (600 MHz, D_2O)

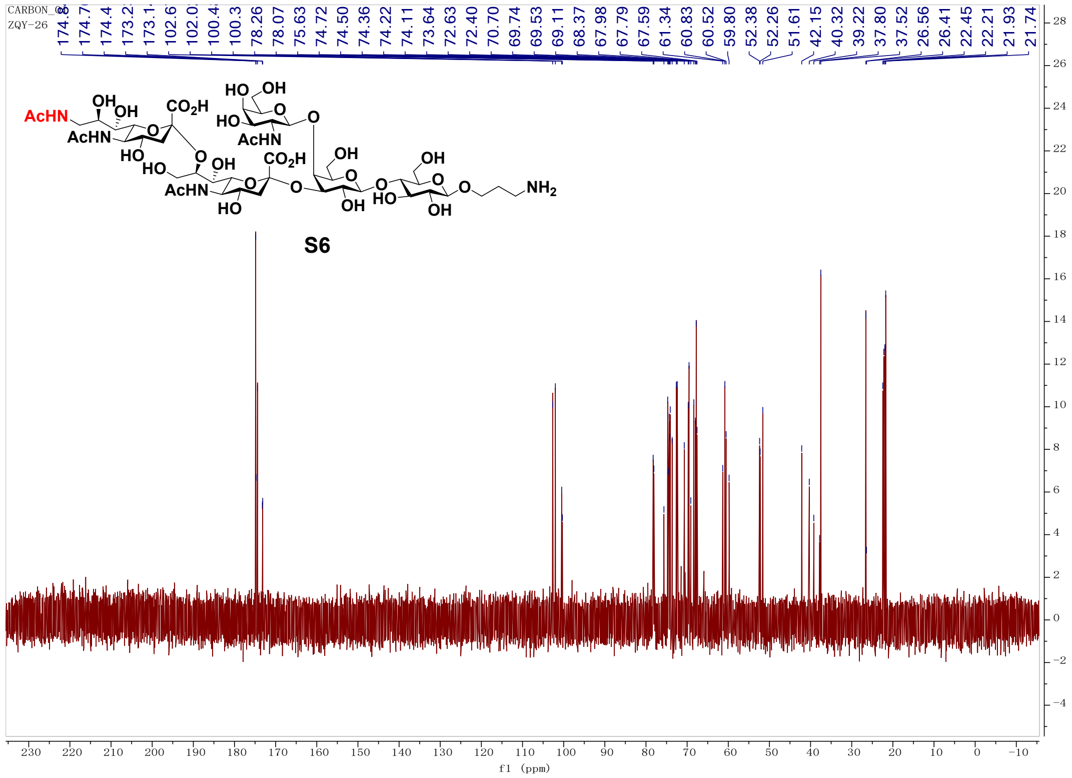

^{13}C-NMR of **S6** (150 MHz, D_2O)

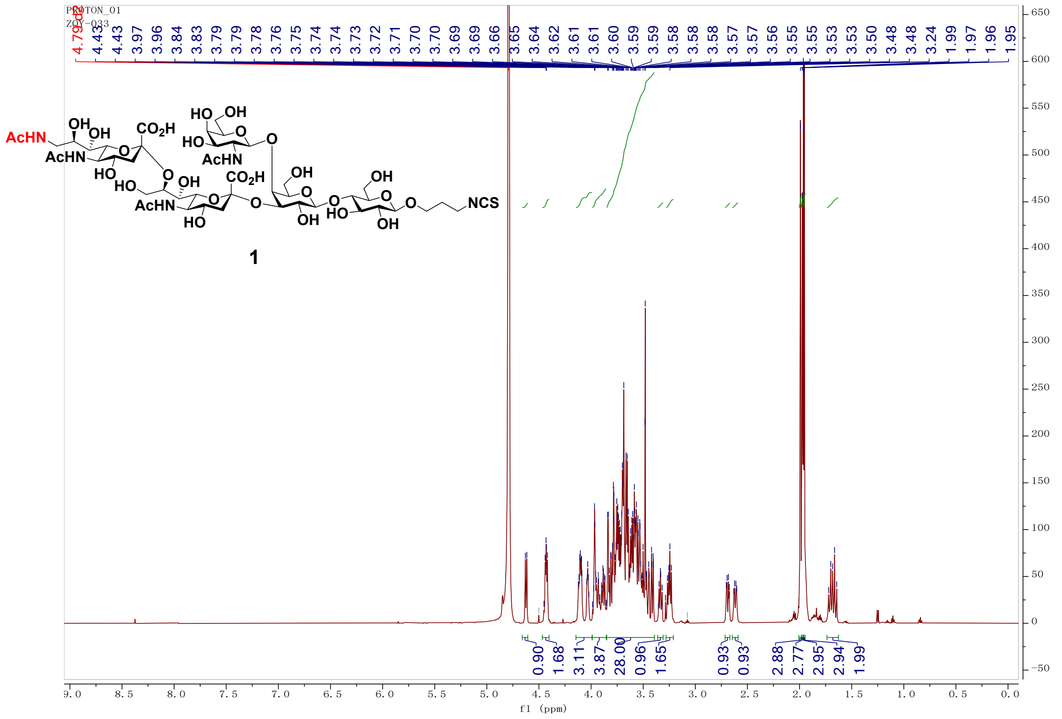

^{1}H-NMR of **1** (600 MHz, D_2O)

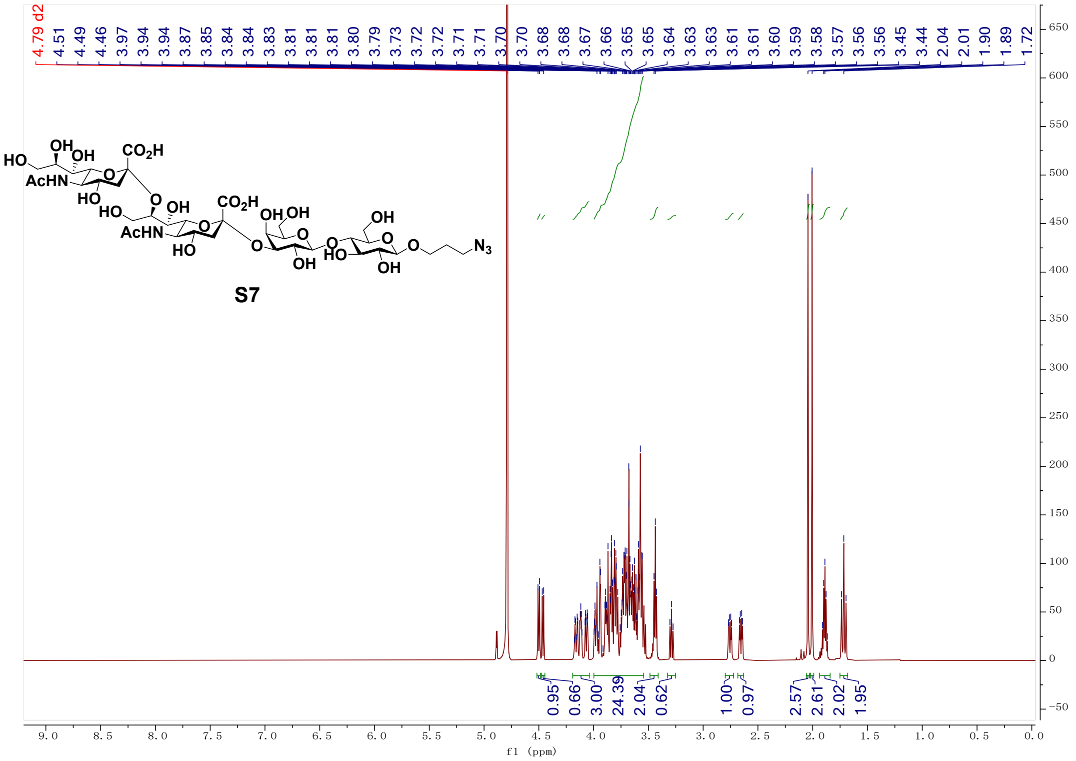

^{1}H-NMR of **S7** (600 MHz, D_2O)

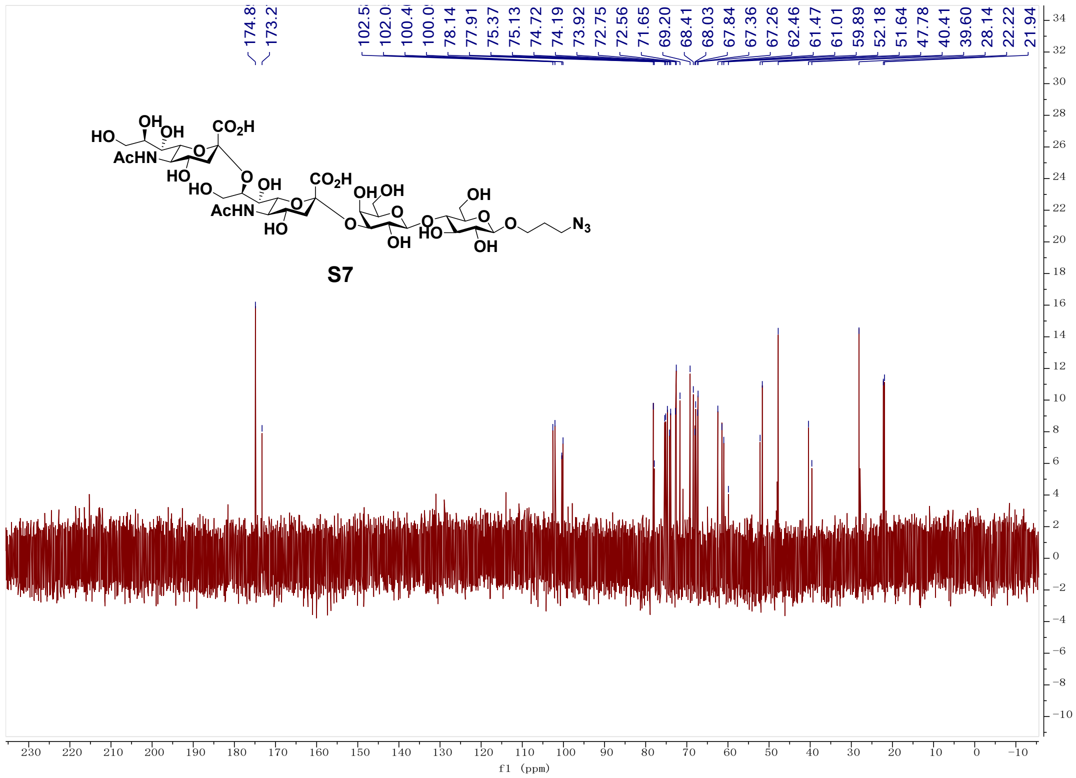

^{13}C-NMR of **S7** (150 MHz, D_2O)

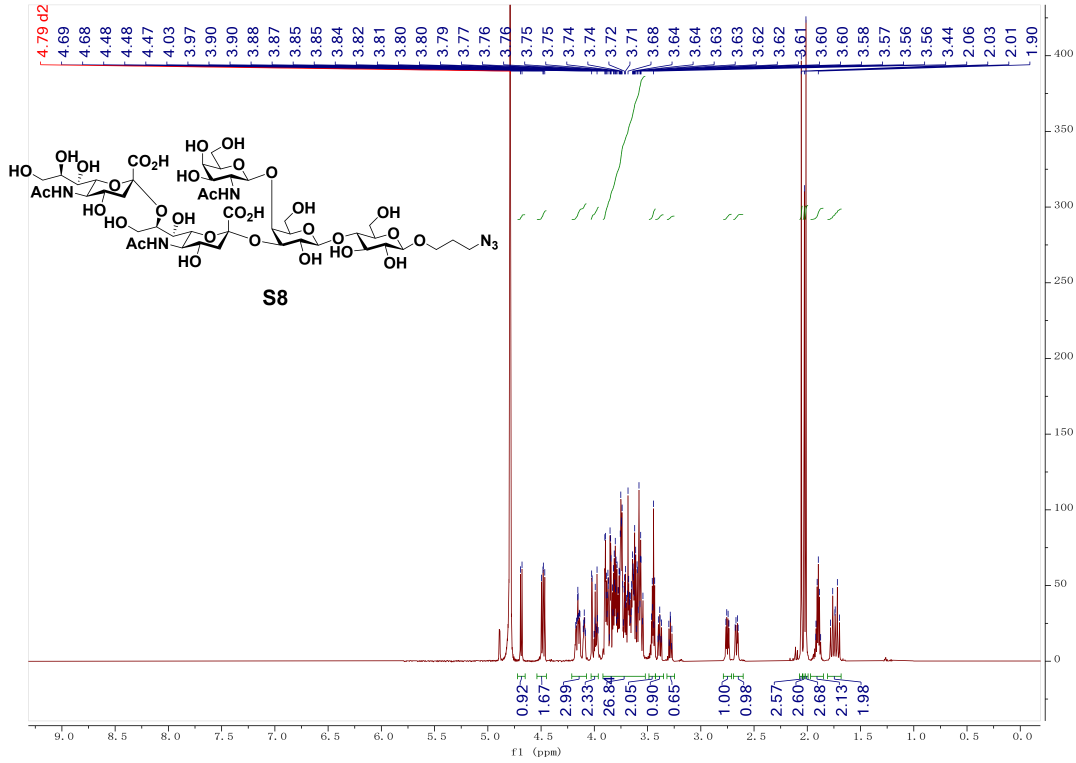

^{1}H-NMR of **S8** (600 MHz, D_2O)

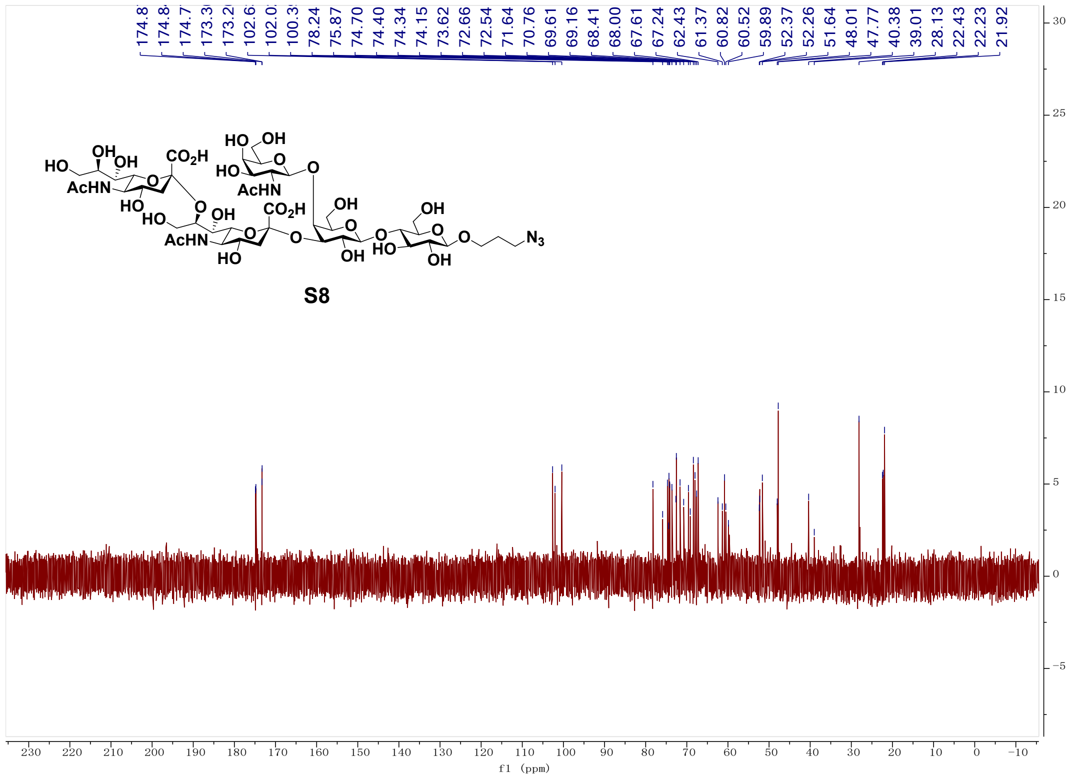

^{13}C-NMR of **S8** (150 MHz, D_2O)

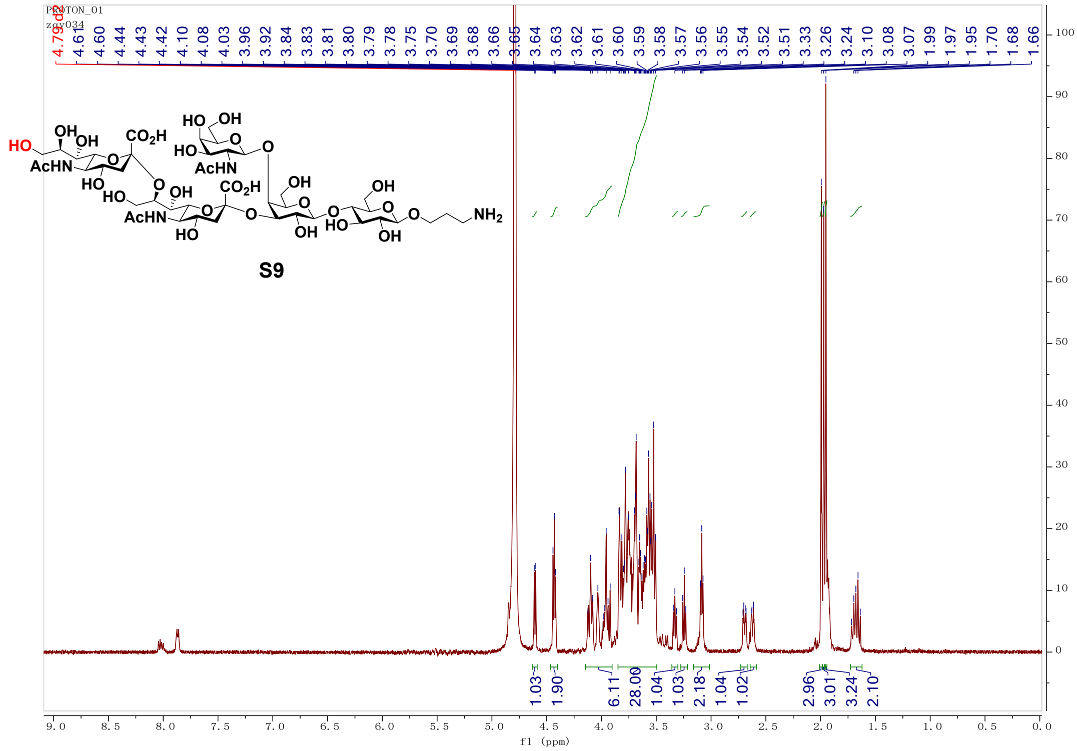

^{1}H-NMR of **S9** (600 MHz, D_2O)

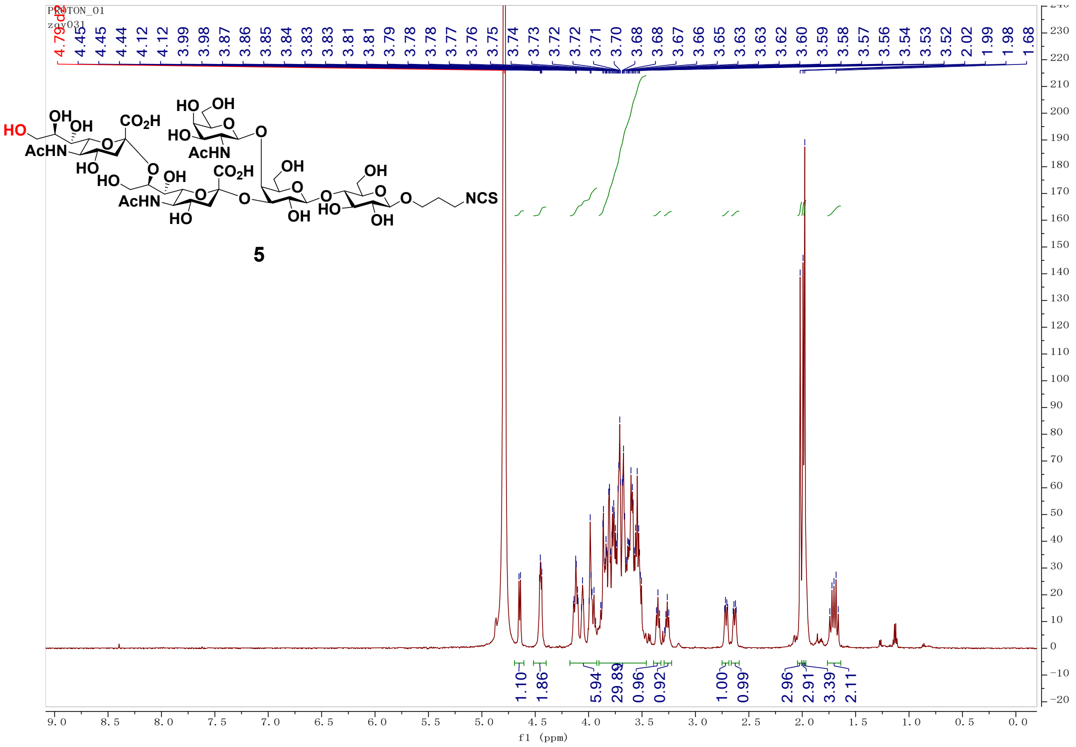

^{1}H-NMR of **5** (600 MHz, D_2O)

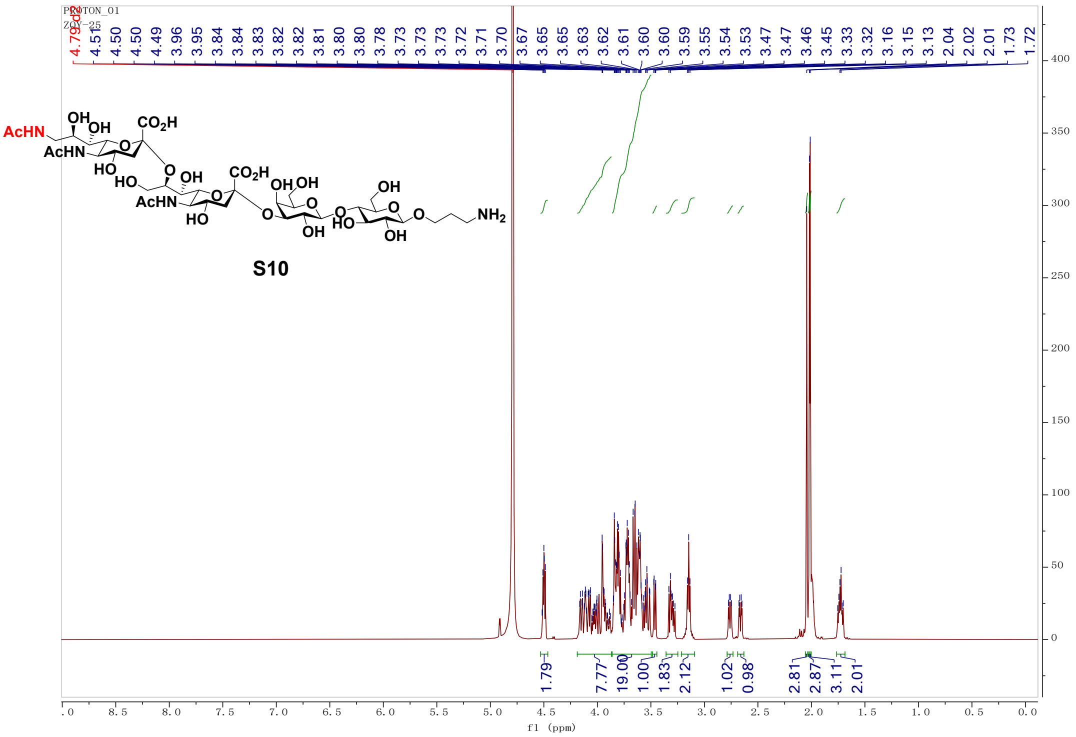

^{1}H-NMR of **S10** (600 MHz, D_2O)

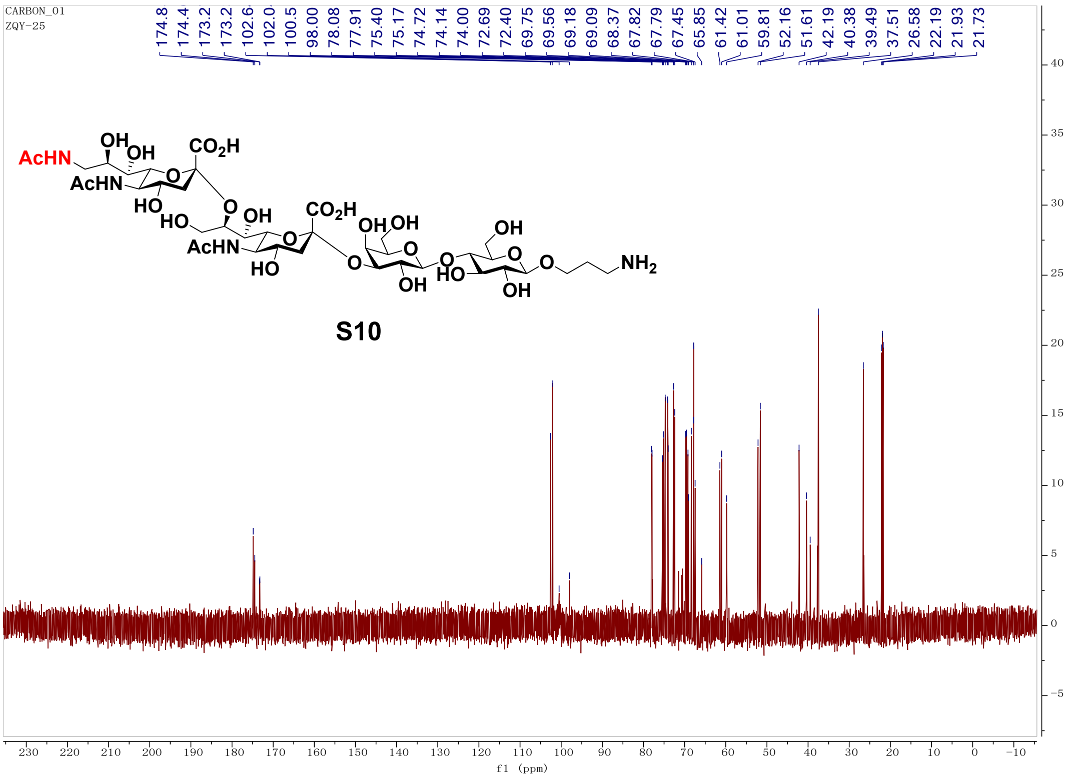

^{13}C-NMR of **S10** (150 MHz, D_2O)

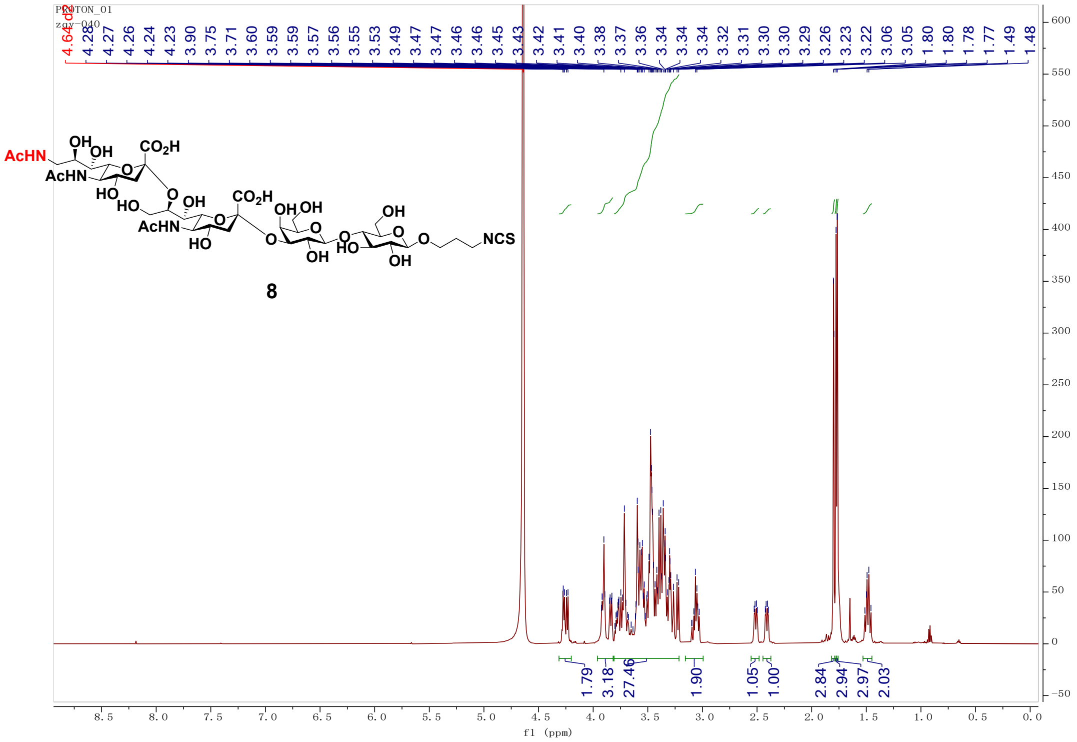

^{1}H-NMR of **8** (600 MHz, D_2O)

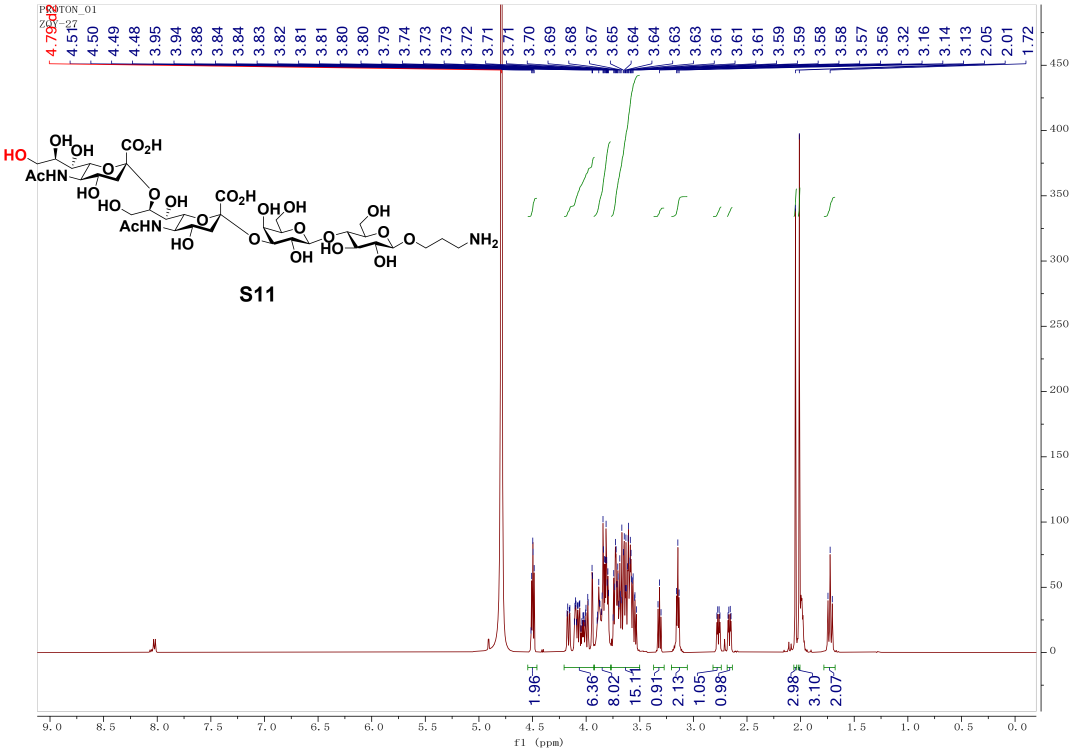

^{1}H-NMR of **S11** (600 MHz, D_2O)

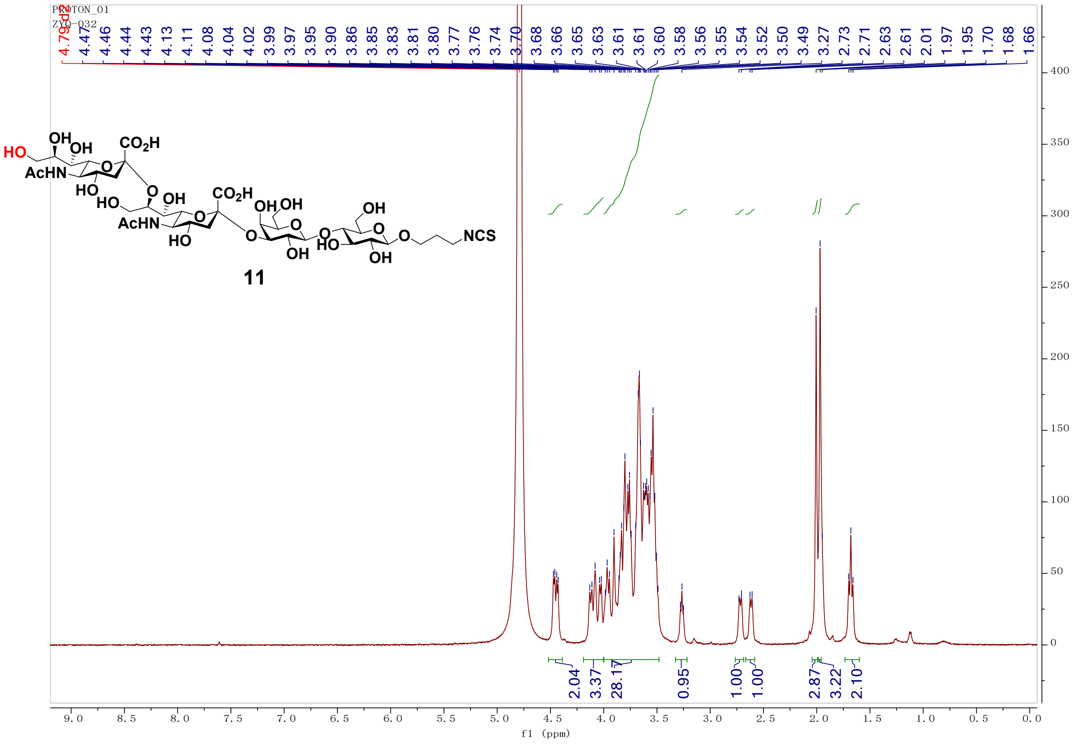

^{1}H-NMR of **11** (600 MHz, D_2O)

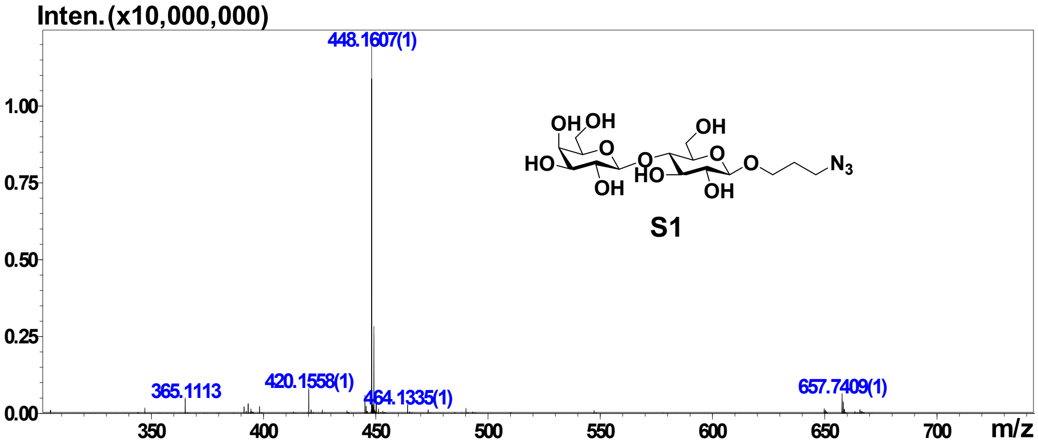

HRMS of **S1**

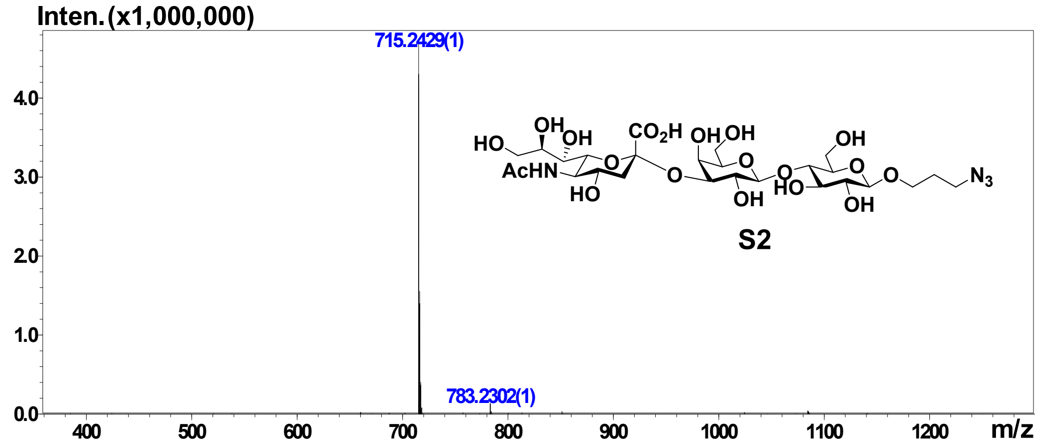

HRMS of **S2**

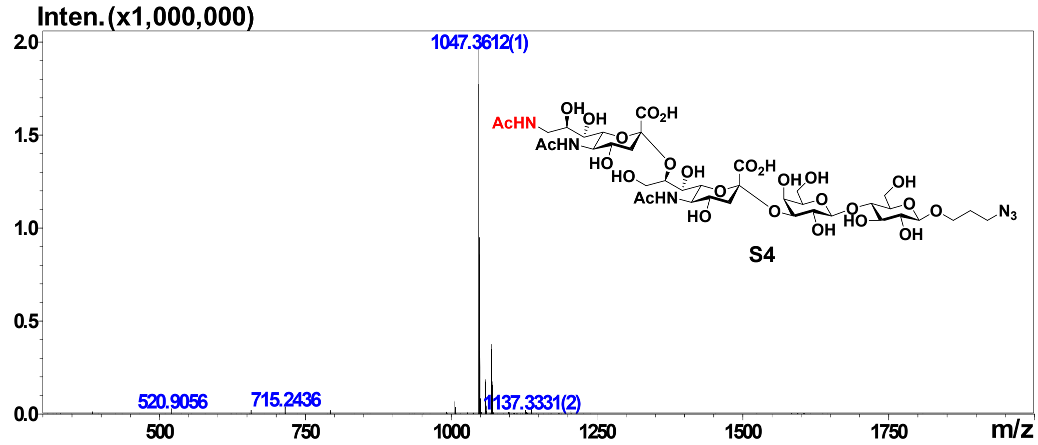

HRMS of **S4**

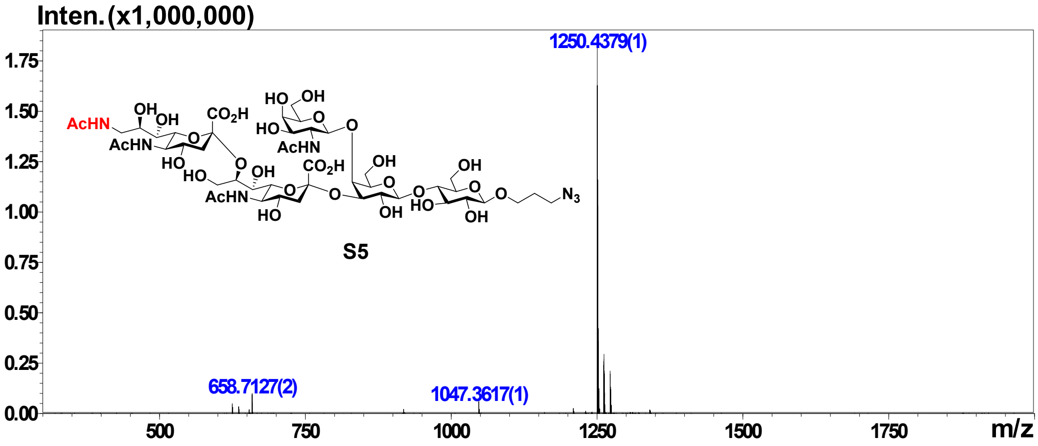

HRMS of **S5**

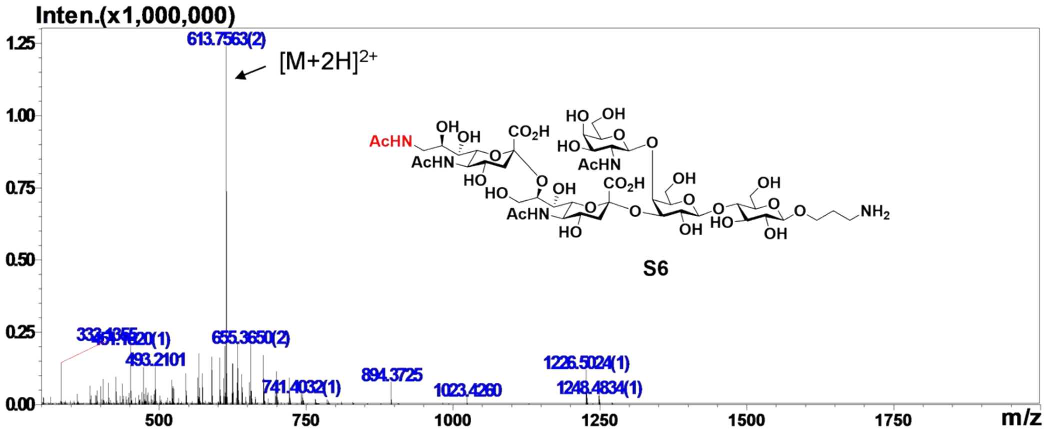

HRMS of **S6**

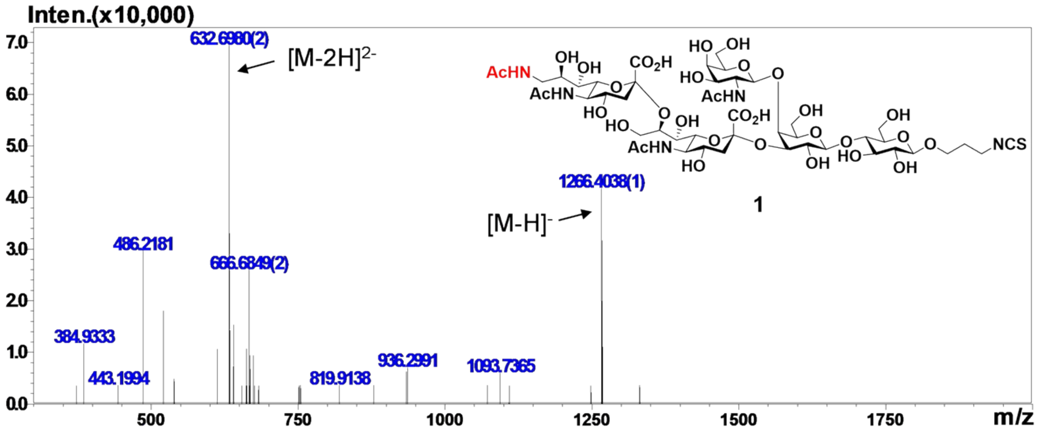

HRMS of **1**

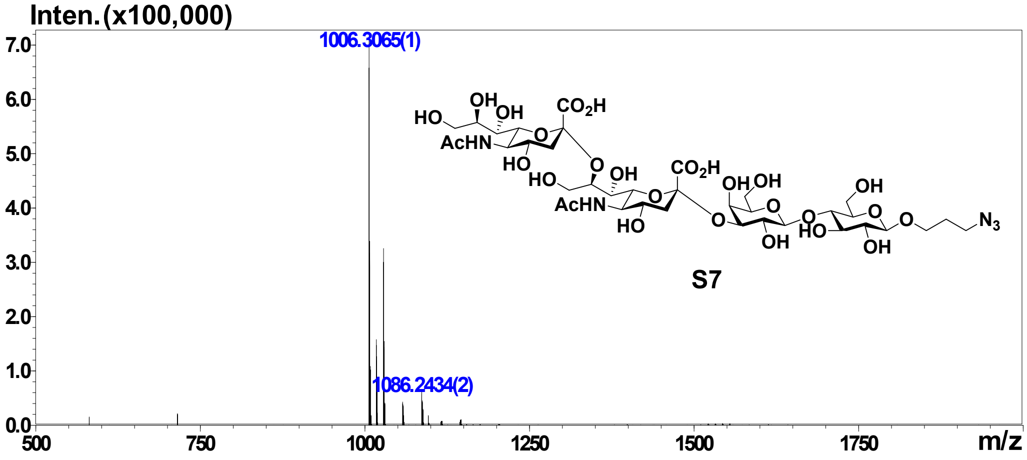

HRMS of **S7**

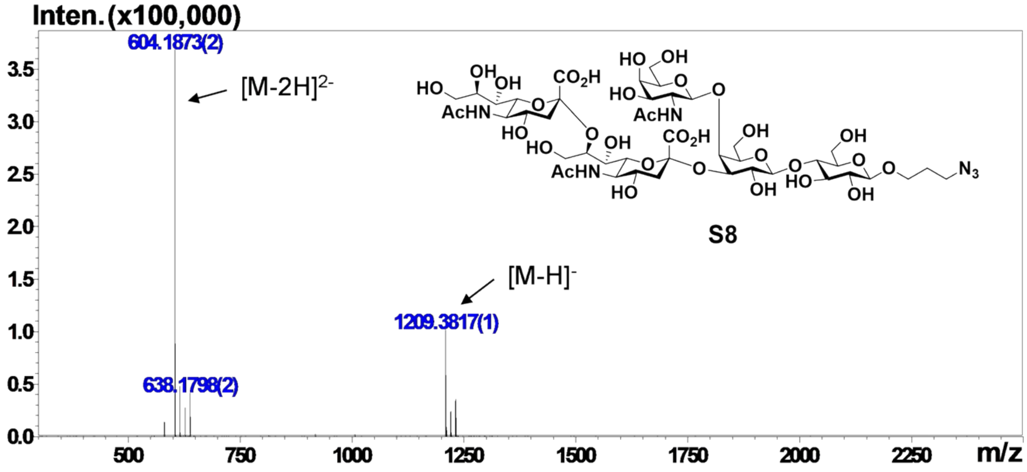

HRMS of **S8**

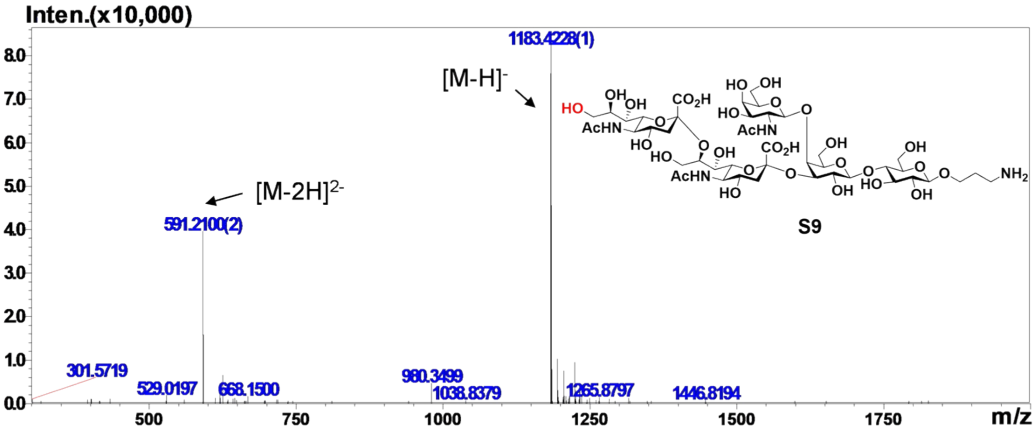

HRMS of **S9**

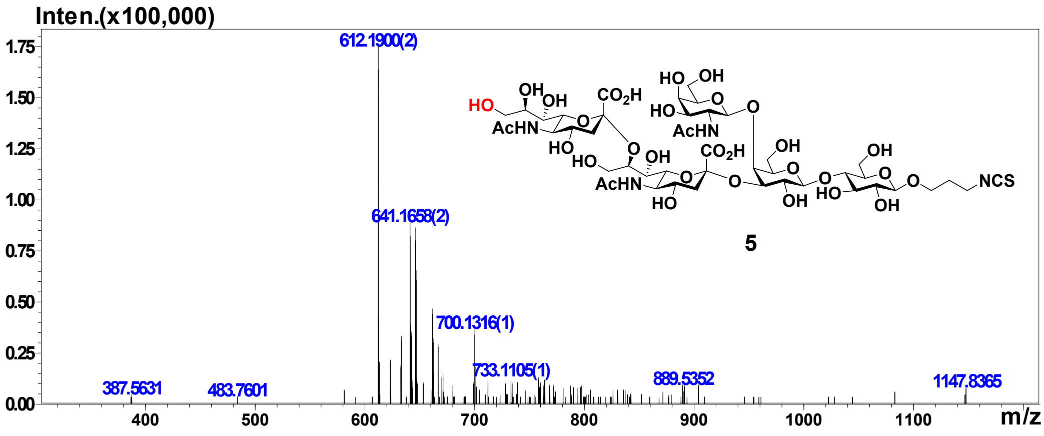

HRMS of **5**

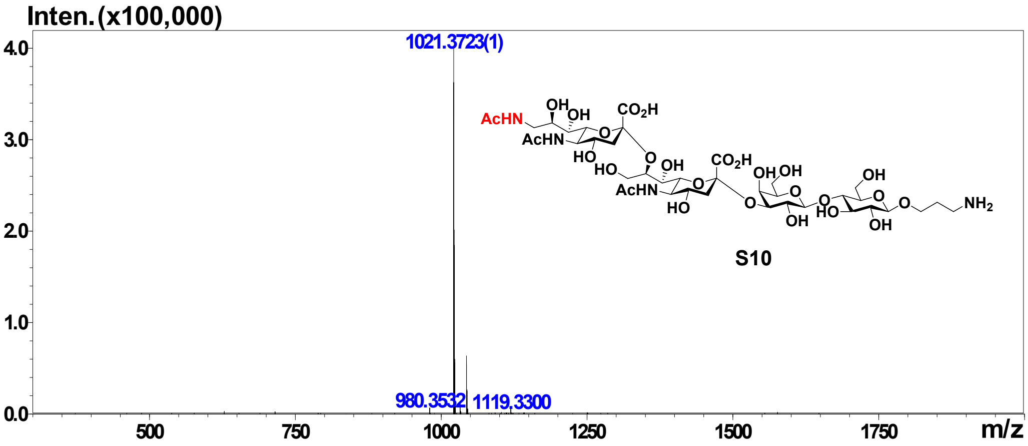

HRMS of **S10**

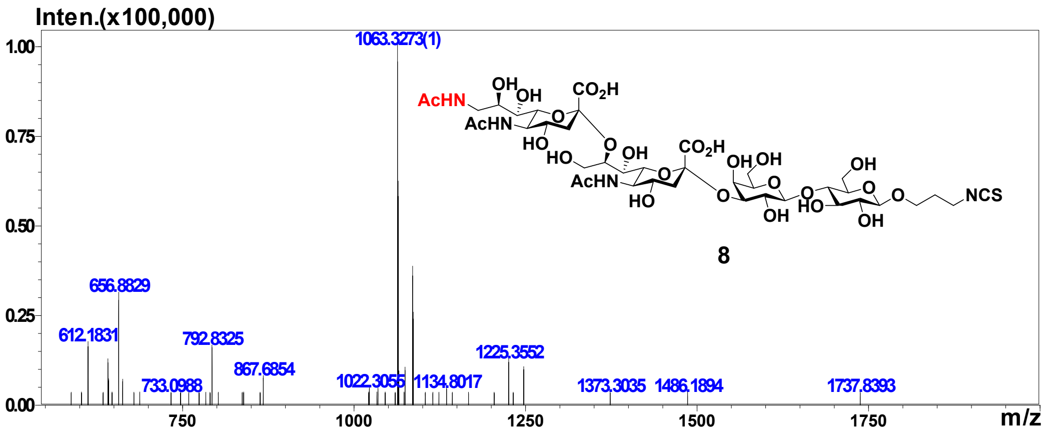

HRMS of **8**

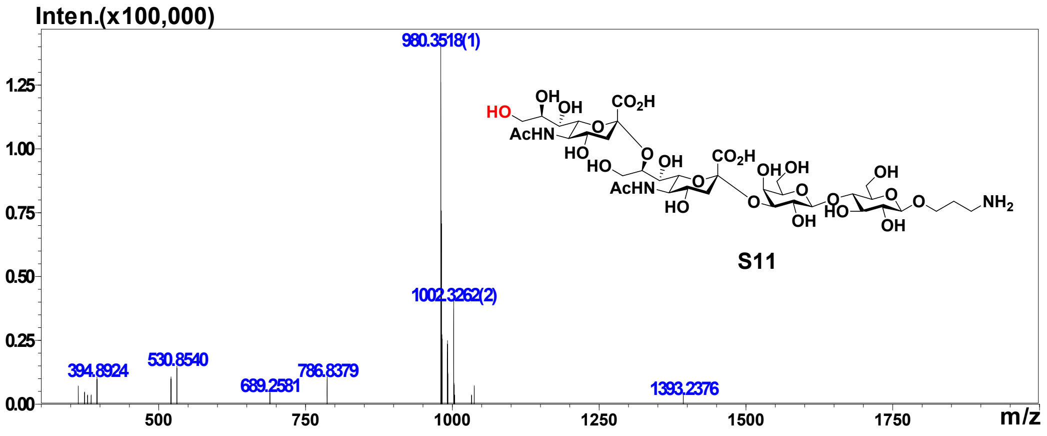

HRMS of **S11**

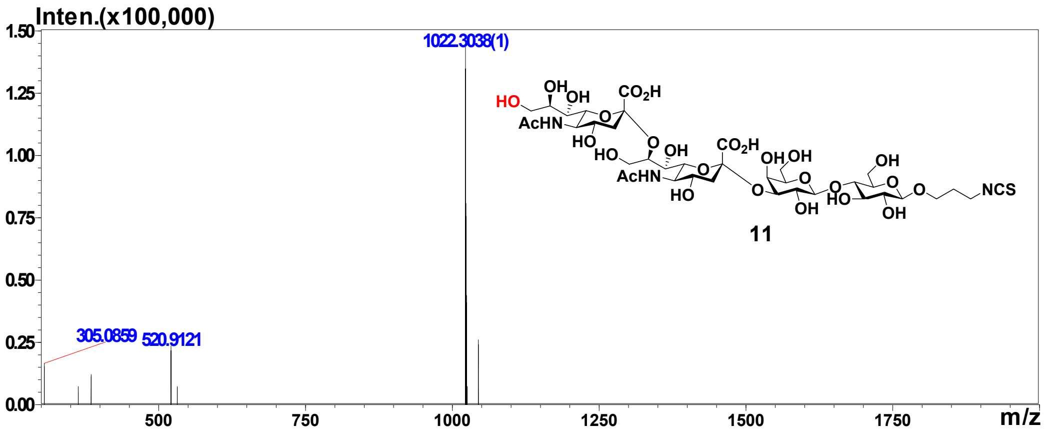

HRMS of **11**

References

1. Y. Li, H. Yu, H. Cao, S. Muthana and X. Chen, *Appl. Microbiol. Biotechnol.*, 2012, **93**, 2411–2423.
2. G. Sugiarto, K. Lau, J. Qu, Y. Li, S. Lim, S. Mu, J. B. Ames, A. J. Fisher and X. Chen, *ACS Chem. Biol.*, 2012, **7**, 1232–1240.
3. H. Yu, J. Cheng, L. Ding, Z. Khedri, Y. Chen, S. Chin, K. Lau, V. K. Tiwari and X. Chen, *J. Am. Chem. Soc.*, 2009, **131**, 18467–18477.
4. H. Yu, Y. Li, J. Zeng, V. Thon, D. M. Nguyen, T. Ly, H. Y. Kuang, A. Ngo and X. Chen, *J. Org. Chem.*, 2016, **81**, 10809–10824.
5. H. Yu, H. Chokhawala, R. Karpel, H. Yu, B. Wu, J. Zhang, Y. Zhang, Q. Jia and X. Chen, *J. Am. Chem. Soc.*, 2005, **127**, 17618–17619.

6. X. Wu, J. Ye, A. T. DeLaitsch, Z. Rashidijahanabad, S. Lang, T. Kakeshpour, Y. Zhao, S. Ramadan, P. V. Saavedra, V. Yuzbasiyan-Gurkan, H. Kavunja, H. Cao, J. C. Gildersleeve and X. Huang, *Angew. Chem. Int. Ed.*, 2021, **60**, 24179–24188.
